# Cardiovascular prescriber attitudes to pharmacogenomics: a survey by the ESC working group on cardiovascular pharmacotherapy

**DOI:** 10.1038/s41397-026-00412-6

**Published:** 2026-04-23

**Authors:** Emma F. Magavern, G.-Andrei Dan, Gianluigi Savarese, Claudio Borghi, Dobramir Dobrev, Juan Tamargo, Peter Ferdinandy, John H. McDermott, William G. Newman, Munir Pirmohamed, Mark J. Caulfield, Juan Carlos Kaski

**Affiliations:** 1 William Harvey Research Institute, Queen Mary University of London, London, EC1M 6BQ UK; 2 Carol Davila University of Medicine, Academy of Romanian Scientists, Bucharest, Romania; 3 Department of Clinical Science and Education, Södersjukhuset; Karolinska Institute, Stockholm, Sweden; 4 Department of Cardiovascular Medicine, University of Bologna-IRCCS AOU S. Orsola, Bologna, Italy; 5 Institute of Pharmacology, West German Heart and Vascular Center, University Duisburg-Essen, Duisburg, Germany; 6 Department of Medicine and Research Center, Montreal Heart Institute and Université de Montréal, Montreal, Canada; 7 Department of Integrative Physiology, Baylor College of Medicine, Houston, TX USA; 8 Department of Pharmacology and Toxicology, School of Medicine, Universidad Complutense, Madrid, Spain; 9 Department of Pharmacology and Pharmacotherapy, Semmelweis University, Budapest, Hungary; 10 Center for Pharmacology and Drug Research and Development, Semmelweis University, Budapest, Hungary; 11 Pharmahungary Group, Szeged, Hungary; 12 Division of Evolution, Infection and Genomics, School of Biological Sciences, University of Manchester, Manchester, M13 9PT UK; 13 Manchester Centre for Genomic Medicine, St Mary’s Hospital, Manchester University NHS Foundation Trust, Manchester, M13 9WL UK; 14 Department of Pharmacology and Therapeutics, Institute of Systems, Molecular and Integrative Biology (ISMIB), University of Liverpool, Liverpool, L69 3GL UK; 15 Cardiovascular and Genomics Research Institute, St George’s, University of London, London, SW18 0RE UK

**Keywords:** Health services, Therapeutics

## Abstract

The aim of this study was to elucidate cardiovascular prescriber access, uptake, and attitudes toward *CYP2C19* and *CYP2D6* genetic testing to guide prescribing of commonly used medications such as clopidogrel, antiarrhythmics, proton pump inhibitors, and antidepressants. A survey, designed in collaboration with the European Society of Cardiology (ESC) WG on Cardiovascular Pharmacotherapy and external experts was disseminated to ESC members using SurveyMonkey. 265 prescribers from 68 countries participated. Most respondents thought testing would be beneficial, though *CYP2C19* testing was perceived as more beneficial (73%) and desirable than *CYP2D6* (61%). Access to *CYP2C19* testing was more common (30%) than *CYP2D6* testing (19%), but mostly outside of public funded health systems. Uptake in those who had access was higher for *CYP2C19* (67%), than for *CYP2D6* (33%). Confidence in interpreting results to prescribe was also higher with *CYP2C19* (69%) than with *CYP2D6* (53%), but most respondents wanted information prior to prescribing. One third of respondents highlighted the need for a turnaround time that matched their clinical practice. Unsolicited Pharmacogenomic (PGx) information from a patient was uncommon, but most prescribers acted on the information. A minority of respondents had undertaken PGx testing themselves, but most wanted testing for relevant medications. Respondents’ experiences as patients made them more likely to believe that PGx testing was warranted. A minority ( ~ 15%) were aware of either local prescriber guidance or patient information materials regarding PGx testing. Prescribers want access to pharmacogenomics data regarding CYP2C19 and CYP2D6 for prescribing cardiovascular medicines. However, there are barriers which hamper implementation. Prescribers lived experience with medication use as patients impacted their views of PGx.

**Figure:**
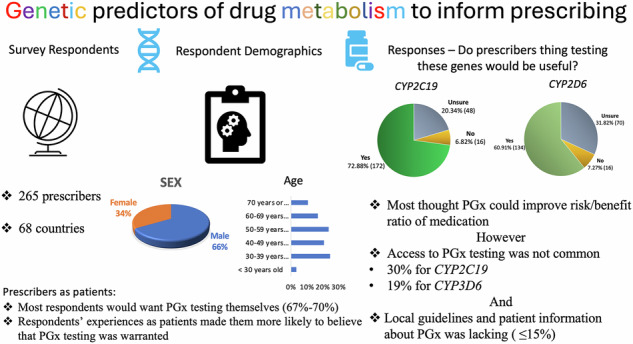
265 prescribers responded to the ESC survey from 68 countries. Most prescribers wanted access to pharmacogenomic testing for *CYP2C19* and *CYP2D6* for their patients and for themselves. Though most prescribers thought these pharmacogenomic tests would be useful and could improve the risk/benefit profile of relevant medications, prescribers responded more positively to *CYP2C19* compared with *CYP2D6* testing. Guidance and information for both prescribers and patients were lacking.

265 prescribers responded to the ESC survey from 68 countries. Most prescribers wanted access to pharmacogenomic testing for *CYP2C19* and *CYP2D6* for their patients and for themselves. Though most prescribers thought these pharmacogenomic tests would be useful and could improve the risk/benefit profile of relevant medications, prescribers responded more positively to *CYP2C19* compared with *CYP2D6* testing. Guidance and information for both prescribers and patients were lacking.

## Introduction

Genetic variation is known to explain some of the interindividual variability in medication response [1]. The prospective multicentre pharmacogenomics (PGx) PREPARE trial showed that prospective genotyping of 12 pharmacogenes can reduce adverse drug reactions by up to 30% [2]. Among these were two key genes - *CYP2C19* and *CYP2D6-* which encode enzymes known to play a key role in the metabolism of several medications (Cytochrome P450 2C19 (CYP2C19 and Cytochrome P450 2D6 (CYP2D6)). *CYP2C19*, the first pharmacokinetic pharmacogene with broad application to be widely used across specialties is now being introduced by regulators before prescribing mavacamten, and it is also being recommended to guide antiplatelet therapy in stroke patients in the UK [3, 4]. However, cardiology as a speciality has been slow to embrace pharmacogenomics, and access to *CYP2C19* genotyping to guide cardiovascular therapies is patchy across Europe. It is only recently that large professional cardiovascular societies have issued position statements and scientific statements supporting the use of *CYP2C19* genotype testing in clinical care [5–7]. There has been extremely limited incorporation of PGx in existing European Society of Cardiology (ESC) clinical guidelines [8]. There has not been a systematic evaluation of access, uptake, or Healthcare Practicioner (HCP) and patient supporting guidance and resources [9]. *CYP2D6* is the gene encoding the CYP2D6 enzyme which is responsible for metabolising many medications commonly used in cardiovascular clinical practice (e.g. metoprolol, flecainide) and approximately one quarter of all pharmacological agents used worldwide (including, among others, codeine, ondansetron, and many antidepressants and antipsychotics) [10]. The *CYP2D6* gene is highly polymorphic, meaning that there are many common and rare variants in this gene, which can impact on how patients metabolise medications [10, 11]. However, prescribing based on knowledge of these variants is not common practice.

To better understand access, uptake, and attitudes toward *CYP2C19* and *CYP2D6* genetic testing to guide prescribing, the ESC Working Group (WG) on Cardiovascular Pharmacotherapy (CVP) invited cardiovascular prescribers to answer a survey on their prescribing and personal experience with *CYP2C19* and *CYP2D6* genetic testing.

## Methods

A survey titled “*Genetic predictors of drug metabolism to inform cardiovascular prescribing*” was designed as a collaborative project with the ESC WG on CVP and external experts. The survey can be viewed in the supplementary materials (Supplement 1). The ESC marketing and communication team optimised question format for clarity and to minimise bias. The SurveyMonkey platform was used to disseminate the survey.

ESC members who self-identified as physicians and had opted in to receiving e-mails from ESC were e-mailed an invitation to complete the survey via an online link (155,873 recipients, including 102,763 Physician cardiologists, 52,586 Physician non-cardiologists and 524 CVP WG members).

Invitation was also disseminated via ESC newsletters and bulletins of the various working groups and associations. Participants were required to provide informed consent to the study to complete the survey. Following consent, none of the survey questions were mandatory to proceed with the survey. The survey was published on April 29th, left open for 4 months, and closed when a month had passed without further responses. All the responses were anonymous.

### Analysis

Descriptive statistics were used to summarize survey responses. Multivariable logistic regression was performed to test the association between personal medication experience and PGx attitudes, adjusting for gender and age range as covariates to control for potential confounding. Statistical analyses were performed using R studio [12]. Confidence intervals were set to 95%.

## Results

There were 290 responses to the survey; 265 identified as prescribers. Non prescribers were not offered the subsequent survey questions. Respondents were working in 68 different countries (*n* = 202 respondents identified their country). The most frequently listed countries were Greece (*n* = 12), Italy (*n* = 11), the United Kingdom (*n* = 11), and Spain (*n* = 9). Most respondents (71%) were physicians specialising in cardiology. Demographics are shown in Table 1. 67% of respondents were male and they spanned a wide age range: 3% < 30 years old, 25% 30–39 years old, 21% 40–49 years old, 24% 50–59 years old, 17% 60–69 years old, and 11% 70 years old or older. 67% of respondents identified themselves as white/caucasian. A full copy of the survey summary results is available in the supplementary materials (Supplement 2).

**Table 1:** Respondent demographics.

Sex *n* = 206	66.5% Male
Age *n* = 207	3.4% Younger than 30 years old
	24.6% 30–39 years old
	20.8% 40–49 years old
	23.7% 50–59 years old
	16.9% 60–69 years old
	10.6% 70 years or older
Professional Role *n* = 209	47.7% Physician – General Cardiology
	30.1% Physician – Sub-specialty in cardiology (heart failure, arrythmia, imaging, acute, prevention)
	11.5% Physician – Internal Medicine
	4.8% Physician – General Practice
	3.8% Other physician
	1.0% Nurse / Advanced Nurse Practitioner
	1.9% Scientist / researcher
	6.2% Other
Race/Ethnicity *n* = 208	66.8% White / Caucasian
	15.4% Asian / Pacific Islander
	5.8% Hispanic
	1.0% Black or African American
	0.5% American Indian or Alaskan Native
	1.9% Multiple ethnicity
	5.8% Other
	2.9% Prefer not to answer

### Prescriber experience with and attitudes toward *CYP2C19* genetic testing

**Fig. 1:**
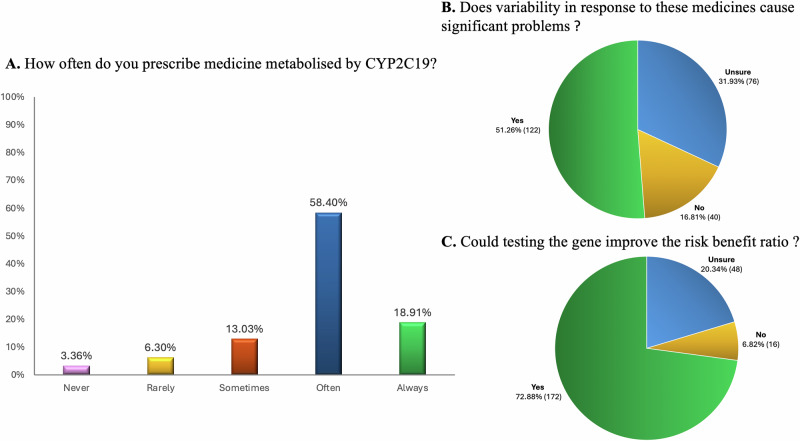
Prescriber experience with and attitudes toward personalising prescribing for CYP2C19 metabolised medications. **A, B, C**. **A** Responses to “How often do you prescribe medicine metabolised by the Cytochrome P450 2C19 (CYP2C19) enzyme (examples include clopidogrel, mavacamten, proton pump inhibitors, tricyclic antidepressants, citalopram, sertraline)?” N 238 responses. **B** Responses to “Do you think that variability in response to these medicines metabolised by CYP2C19 causes significant problems for your patients?” N 238 responses. **C** Responses to “Do you think that testing the *CYP2C19* gene, which encodes the CYP2C19 enzyme, could improve the risk benefit ratio of at least one CYP2C19 metabolised medicine for your patients?”.

77% of respondents reported often or always prescribing medicine metabolised by the CYP2C19 enzyme (examples given included clopidogrel, mavacamten, proton pump inhibitors, tricyclic antidepressants, citalopram, and sertraline) (Fig. 1A), and 51% of respondents thought that variability in response to these medications causes significant problems for their patients (Fig. 1B). 73% thought that testing for the *CYP2C19* gene could improve the risk benefit ratio of at least one CYP2C19 metabolised medicine for their patients (Fig. 1C). Only 30% of respondents had access to *CYP2C19* genetic testing, either through a public health care affiliated laboratory (50% of all those with access) or a private provider (56% of all those with access, including 6% of respondents with public health care affiliated access). 67% of those 72 respondents who did have access to *CYP2C19* genetic testing had ordered a test. 69% of those respondents who had ordered such a test felt confident with interpreting the results to inform prescribing. Only 14% of respondents had ever been presented by a patient with *CYP2C19* genetic testing results that they had not requested. However, for 82% of the respondents who had been presented with this information by a patient, the results changed the respondent’s prescribing choice. 49% of respondents would want to know their patients’ *CYP2C19* genetic testing results before prescribing a medicine metabolised by CYP2C19. An additional 32% would want this information if the turnaround time matched their requirements.

### Prescriber experience with and attitudes toward CYP2D6 genetic testing

**Fig. 2:**
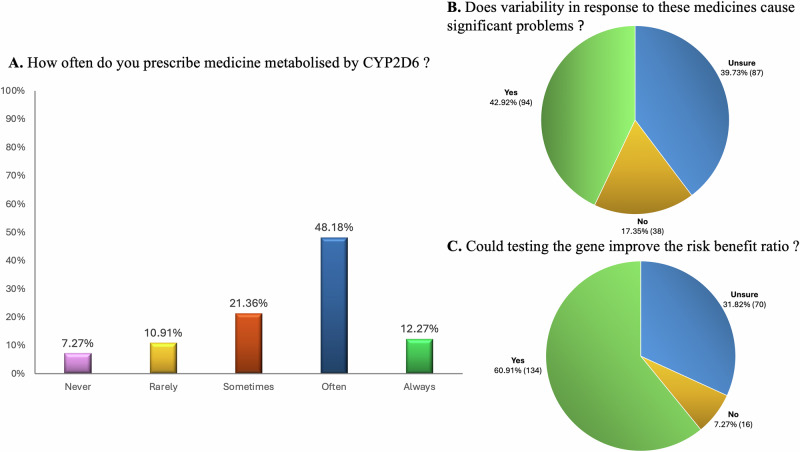
Prescriber experience with and attitudes toward personalising prescribing for CYP2D6 metabolised medications. **A, B, C**. Responses to “How often do you prescribe medicine metabolised by the Cytochrome P450 2D6 (CYP2D6) enzyme (examples include metoprolol, flecainide, ondansetron, codeine, tramadol, tamoxifen, paroxetine, some tricyclic antidepressants, many antipsychotics)?” N 220 responses. **B** Responses to “Do you think that variability in response to these medicines metabolised by CYP2D6 causes significant problems for your patients?” N 219 responses. **C** Responses to “Do you think that testing the *CYP2D6* gene, which encodes the CYP2D6 enzyme, could improve the risk benefit ratio of at least one CYP2D6 metabolised medicine for your patients?”. N 220 responses.

60% of respondents reported often or always prescribing medicine metabolised by the CYP2D6 enzyme (examples given included metoprolol, flecainide, ondansetron, codeine, tramadol, tamoxifen, paroxetine, some tricyclic antidepressants, many antipsychotics) and 43% of respondents thought that variability in response to these medications causes significant problems for their patients (Fig. 2A & B). 61% thought that testing the *CYP2D6* gene could improve the risk benefit ratio of at least one CYP2D6 metabolised medicine for their patients (Fig. 2C). Only 19% of respondents had access to *CYP2D6* genetic testing, either through a public health care affiliated laboratory (61% of all those with access) or a private provider (49% of all those with access). 33% of those 42 respondents who did have access to *CYP2D6* genetic testing had ordered a test. 53% of those respondents who had ordered such a test felt confident with interpreting the results to inform prescribing. Only 8% of respondents had ever been presented with *CYP2D6* genetic testing results that they had not requested by a patient. However, for 72% of the respondents who had been presented with this information by a patient the results changed the respondent’s prescribing choice. 46% of respondents would want to know their patients’ *CYP2D6* genetic testing results before prescribing a medicine metabolised by CYP2D6. An additional 31% would want this information if the turnaround time matched their requirements.

### Reported access to testing by country of employment

*CYP2C19* testing access was reported by few individual respondents across many countries with a maximum threshold of 5 individuals per country reporting access in The Netherlands (with public access) or Greece (with private access). For *CYP2D6* testing access, The Netherlands respondents reported the most frequent access, with only 4 respondents reporting access via the public health system. A maximum of 1–2 respondents from any given country reported private laboratory access to *CYP2D6* testing.

### Prescriber access to relevant PGx clinical decision support information and patient information resources

12% and 8% of respondents respectively were aware of local (institutional or national) guidance to action *CYP2C19* or *CYP2D6* genetic testing results. Only 15% and 11% of respondents respectively were aware of having access to institutionally, nationally, or internationally standardised written patient education material for patients regarding *CYP2C19* or *CYP2D6* testing.

### Prescriber perspective on the role of PGx in Drug-Drug interactions and health equality

69% of respondents thought that the magnitude of drug-drug interactions can be modified by the *CYP2C19* or *CYP2D6* genotype, while 81% thought that the magnitude of drug-drug interactions can be modified by a medical comorbidity such as obesity, diabetes, liver disease or renal impairment. 73% of respondents thought that *CYP2C19* or *CYP2D6* genotyping would improve health equality, and 52% thought that ancestry impacts on *CYP2C19* or *CYP2D6* genotype.

### Prescribers’ personal experience with PGx medications and PGx preferences

41% of respondents reported having taken a medication metabolised by CYP2C19, while 35% reported having taken a medication metabolised by CYP2D6. 12% of respondents had undertaken *CYP2C19* testing and 10% had undertaken *CYP2D6* testing. However, 70% and 67% of respondents respectively stated that they would like to be offered a *CYP2C19* or *CYP2D6* test if they had an indication to receive a relevant medication. 35% and 30% of respondents respectively stated that they would pay for a private *CYP2C19* or *CYP2D6* test. Of those individuals who said they would pay for a private test, the average maximum value that respondents would be willing to pay for such a private test was 121 Euros (Median 75 Euros, range 0–2000 Euros).

### Perception of medication adherence and impact of PGx testing on adherence

Respondents thought that their patients were on average 68% (standard deviation +/−17%, *n* = 205) compliant with medication. 50% of respondents thought that testing the *CYP2C19* gene could improve medication adherence, while 43% of respondents thought that testing the *CYP2D6* gene could improve medication adherence.

### Relationship between personal experience and PGx attitudes

Respondents who had themselves taken a medicine metabolised by CYP2C19 were more likely to report that testing the *CYP2C19* gene could improve the risk benefit ratio of at least one relevant medication for their patients (odds ratio (OR) 2.3 confidence interval (CI) 1.2–4.7, *p* 0.022). The same relationship held true for association between respondent experience with CYP2D6 metabolised medication and perception that testing the *CYP2D6* gene could improve the risk benefit ratio of at least one relevant medication for their patients (OR 3.4 CI 1.8–6.8, *p* < 0.001).

Those practitioners who would like to be offered *CYP2C19* genetic testing if they had an indication to receive a medication metabolised by CYP2C19 were more likely to perceive the *CYP2C19* gene testing as useful to their patients (OR 10.0, CI 4.9–20.1, *p* < 0.001). The same was true regarding an association between desire for personal access to *CYP2D6* genetic testing and perception of *CYP2D6* genetic testing utility for patients (OR 10.9, CI 5.5–22.9, *p* < 0.001).

## Discussion

This study is the first to work with an international professional body to assess cardiovascular prescriber access to PGx testing, clinical decision support information, patient information and personal use of PGx for two key drug metabolism pharmacogenes with implications for multiple medications across the cardiovascular and non-cardiovascular prescribing spectrum.

Though most respondents commonly prescribed medicines metabolised by both CYP2C19 and CYP2D6, fewer respondents reported prescribing medications metabolized by CYP2D6 as opposed to those metabolized by CYP2C19. Half or less of all respondents perceived variability in response to these medications as problematic. Testing *CYP2D6* was perceived as beneficial and desirable by fewer respondents as compared with *CYP2C19* testing, though most respondents thought that testing for both would be beneficial for their patients. It is interesting that fewer respondents thought variability in medication response was problematic (51% for CYP2C19, 43% for CYP2D6)) than thought that genetic testing could improve the risk benefit ratio of at least one relevant medicine for their patients (73% *CYP2C19*, 61% *CYP2D6*). While most respondents thought that PGx would improve health equality, the fact that only approximately one in every two thought that ancestry had an impact on genotype leaves some unanswered questions around what other pathways prescribers perceived PGx to impact on health equality. This should be explored in future studies.

### Access to PGx testing

Most respondents did not have access to PGx testing. Access to *CYP2C19* testing was more common than access to *CYP2D6* testing, which may be a result of the high strength of evidence associating CYP2C19 with response to clopidogrel and the technical ease of identifying genetically predicted metaboliser state of CYP2C19 as compared with CYP2D6. Half of that access was outside of public funded health systems, showing the role that private health care and industry is currently occupying in PGx as compared with other areas of medicine. There was not a high enough volume of respondents from any individual country to assess consistency of access within and between countries.

### PGx testing uptake and interpretation

Uptake of pharmacogenomic testing by those prescribers who had access was higher with *CYP2C19*, with most prescribers (67%) ordering tests, than it was with *CYP2D6*, with most prescribers not ordering these tests (33% uptake). This seems appropriate given the relative strength of evidence for PGx testing of medications prescribed by Cardiologists.

Confidence in interpreting results to prescribe was also higher with *CYP2C19* results (69%) than with *CYP2D6* results (53%).

### Unrequested PGx results

There has been discussion in the literature around clinicians being presented with PGx tests results that a patient has received from direct-to-consumer services, and increasingly research studies are returning PGx results directly to patients [13–15]. Most respondents had not experienced this, with only a minority reporting receiving this information from a patient. It is interesting that with some apprehension about this scenario conveyed by the literature, most respondents who did encounter this unsolicited information changed their prescription because of the information. This may be a select group of patients and prescribers, or it may signal that this information would be utilised at high rates if it were available, even if the clinician would not have chosen to request it. There are a range of consortia guidelines available to support clinicians at the national and international level, though they do not provide information on testing indications and may be informed by local prescribing patterns that are less relevant to other prescribing contexts. The most recognised guidelines come from the Clinical Pharmacogenetics Implementation Consortium and the Dutch Pharmacogenetics Working Group [16, 17]. The Pharmacogenomics Knowledgebase (PharmGKB) is funded by the USA National Institute of Health and is a vital resource integrating the available evidence and guidelines [18].

### Desirability of PGx information

Most respondents wanted information about genetic variants in *CYP2C19* and *CYP2D6* prior to prescribing a relevant medication, with approximately a third of respondents qualifying that with a requirement for a turnaround time that matched their needs. This finding highlights the importance of the clinical implementation pathway around testing, and the need to tailor choice of technology and process to clinical needs [19].

### Availability of educational materials for HCPs and patients

Lack of HCP PGx knowledge has been highlighted previously, as has poor patient understanding of PGx results [20, 21]. Given these gaps and the above desirability of PGx testing from prescribers, clinical decision support guidance for prescribers and patient information materials are both critical to appropriate implementation initiatives, yet we are unaware of a broad initiative to collate availability of such resources across international prescribers. The results of this study, with 15% or less of respondents aware of either local health care professional (HCP) guidance or patient information materials regarding PGx testing for either *CYP2C19* or *CYP2D6*, highlight an urgent need for support and funding to target these domains.

### Prescribers as patients

Approximately one in three of the respondents had themselves taken a medication metabolised by CYP2D6, with more respondents having used a medication metabolised by CYP2C19. While a minority of respondents had undertaken PGx testing, most wanted to be offered such testing if a relevant medication was indicated in their own treatment. Respondents’ experiences as patients seem to have an impact on their attitudes as prescribers with respondents exposed to a relevant medication more likely to believe that PGx testing for the associated gene could improve the risk benefit ratio. We are not aware of any other studies that have explored this relationship.

### Adherence

Respondents seemed to have a more optimistic view of their patient’s adherence to medication than the literature would suggest, with the average perceived adherence being 68% while the literature has found rates of adherence to be substantially lower [22]. Prescribers didn’t have a clear consensus that adherence could be improved with PGx testing, with 50% or fewer believing this was the case for either of the genes discussed. This uncertainty is consistent with conflicting evidence in the literature regarding PGx potential to impact on medication adherence [23–26].

### Limitations

There are limitations to this study. It had a modest number of responses and may have been impacted by selection bias. It is likely that the respondents had a particular interest in the topic and therefore these results may overestimate prescriber awareness and access to pharmacogenomics testing and guidance and may not be representative of the general cardiovascular prescriber. However, though the number of respondents to this survey was modest, the geographical spread was wide, and therefore this provides a new and important addition to the literature providing insight into the availability of PGx testing and guidance and attitudes of prescribers internationally.

## Conclusions

Prescribers want access to pharmacogenomics data regarding *CYP2C19* and *CYP2D6* but there is a stronger desire for and perceived utility of *CYP2C19* genetic testing as compared with *CYP2D6*. Currently access to testing is uncommonly available and likely to come from the private sector. Awareness of local resources for prescribers and patients to utilise and explain PGx testing is very low, and this represents a significant barrier to pharmacogenomics implementation. Turnaround time of PGx testing was a critical aspect of PGx testing desirability for one in every three prescribers. Prescribers lived experience with medication use as patients impacted their views on the potential of PGx to improve medication risk benefit ratio.

Current guidelines used by most of the invited participants do not offer clear recommendations on PGx testing use. Incorporation of existing guidance in clinical guidelines is an important area for future development and action.

## Key messages

### What is already known on this topic

- Uptake of personalised prescribing using genetics amongst cardiovascular prescribers has been low. It is unclear how many of these healthcare practitioners have or would like access to such genetic tests to guide prescribing.

### What this study adds

- This study shows that prescribers who responded to the European Society of Cardiology (ESC) survey want access to genetic testing to personalise prescribing, but that information about pharmacogenomics was lacking for both prescribers and patients.

### How this study might affect research, practice or policy

- These results show overwhelming support for further implementation of pharmacogenomics in cardiovascular care and may lend member support for further integration of pharmacogenomics in ESC professional guidelines and activities.

## Supplementary information

Supplement 1

Supplement 2

## Data Availability

All relevant data has been made available openly in the supplementary materials provided.
