# Supplementary material for: Cardiovascular prescriber attitudes to pharmacogenomics: a survey by the ESC working group on cardiovascular pharmacotherapy
Source: Pharmacogenomics J. 2026 Apr 23;26(3):17. doi: 10.1038/s41397-026-00412-6 (PMC13106032; doi:10.1038/s41397-026-00412-6)
Supplement: Supplementary file 1 — Supplement 1 [file 41397_2026_412_MOESM1_ESM.pdf]

Thank you for your interest in this study to assess attitudes, availability, and uptake of *CYP2C19* and *CYP2D6* genotyping to inform cardiovascular prescribing. This survey will last 6 minutes.

The prospective multicentre PREPARE trial showed that prospective genotyping of 12 pharmacogenes can reduce adverse drug reactions by 30%. *CYP2C19*, the first pharmacokinetic pharmacogene with broad application across specialties is being introduced by regulators as it is required to prescribe mavacamten and recommended to guide antiplatelet therapy in stroke in the UK. However, access to *CYP2C19* genotyping is variable across Europe and there has not been a systematic evaluation of access, uptake, and HCP and patient supporting guidance and resources. CYP2D6 metabolises 25% of medications and the gene that encodes the enzyme is known to have many common variants that affect function of the enzyme. Testing the *CYP2D6* gene is much more technically difficult than testing *CYP2C19*, and it is unclear how many prescribers have access to this testing. This survey will provide much needed answers to these questions on an international scale.

Please note:

- We will not disclose your identity to any third party.
- We comply with the European General Data Protection Regulation (GDPR) 2016/679. Any personal data processed in connection with this survey will be treated confidentially and only used by the ESC for the purposes of market research and not for promotion. Survey results will be kept for a maximum of 48 months for analysis and quality control purposes. We take all reasonable care to prevent any unauthorised access to your personal data. We respect your privacy and your right to access, modify, or remove your personal data. At any time, you can ask to know what personal data is being held. If you have any questions about data protection or require further information, please contact our data protection officer (DPO) at dpo@escardio.org.
- You have the right to end your participation in this survey at any time.

1. Please confirm whether you have considered all the clauses above and accept to participate in this survey.

○ Yes ○ No

2. Do you prescribe medicines?

○ Yes ○ No

3. How adherent do you think your patients are generally with medication

1 100

CYP2C19 - Prescribing experience

4. How often do you prescribe medicine metabolised by the Cytochrome P450 2C19 (CYP2C19) enzyme (examples include clopidogrel, mavacamten, proton pump inhibitors, tricyclic antidepressants, citalopram, sertraline)?

○ Never ○ Rarely ○ Sometimes ○ Often ○ Always

5. Do you think that variability in response to these medicines metabolised by CYP2C19 causes significant problems for your patients?

○ Yes ○ No ○ Unsure

6. Do you think that testing the *CYP2C19* gene, which encodes the CYP2C19 enzyme, could improve the risk benefit ratio of at least one CYP2C19 metabolised medicine for your patients?

○ Yes ○ No ○ Unsure

7. Do you think testing the *CYP2C19* gene could improve medication adherence?

○ Yes ○ No ○ Unsure

8. Do you have access to *CYP2C19* genetic testing? Tick all that apply

☐ Yes, from a public health care affiliated laboratory

☐ Yes, from a private provider

☐ I do not have access to *CYP2C19* testing

☐ I am not sure if I have access to *CYP2C19* testing

CYP2C19 genetic test

9. Have you ever ordered a *CYP2C19* genetic test?

○ Yes ○ No

10. If you have ordered this *CYP2C19* genetic test, did you feel confident with interpreting the results to inform prescribing?

○ Yes

○ No

○ The testing facility interpreted the results for me

11. Do you have local (institutional or national) guidance to action *CYP2C19* results?

○ Yes ○ No ○ Unsure

12. Have you ever been presented with *CYP2C19* genetic testing results that you did not request from a patient you are treating?

○ Yes ○ No

Change in prescribing choices

13. If you have been presented with *CYP2C19* genetic testing information by a patient, did the genetic information presented change your prescribing choices for this patient?

○ Yes

○ No

14. Would you want to know your patients' *CYP2C19* genetic testing results before prescribing a medicine metabolised by CYP2C19?

- ◯ Yes
- ◯ No
- ◯ I would only want this information if the turnaround time matched my requirements
- ◯ Unsure

15. Do you have access to institutionally, nationally, or internationally standardised written patient education material for patients regarding *CYP2C19* testing?

◯ Yes ◯ No ◯ Unsure

CYP2D6 - Prescribing experience

16. How often do you prescribe medicine metabolised by the Cytochrome P450 2D6 (CYP2D6) enzyme (examples include metoprolol, flecainide, ondansetron, codeine, tramadol, tamoxifen, paroxetine, some tricyclic antidepressants, many antipsychotics)?

◯ Never ◯ Rarely ◯ Sometimes ◯ Often ◯ Always

17. Do you think that variability in response to these medicines metabolised by CYP2D6 causes significant problems for your patients?

◯ Yes ◯ No ◯ Unsure

18. Do you think that testing the *CYP2D6* gene, which encodes the CYP2D6 enzyme, could improve the risk benefit ratio of at least one CYP2D6 metabolised medicine for your patients?

◯ Yes ◯ No ◯ Unsure

19. Do you think testing the *CYP2D6* gene could improve medication adherence?

◯ Yes ◯ No ◯ Unsure

20. Do you have access to *CYP2D6* genetic testing? Tick all that apply

- ☐ Yes, from a public health care affiliated laboratory
- ☐ Yes, from a private provider
- ☐ I do not have access to *CYP2D6* testing
- ☐ I am not sure if I have access to *CYP2D6* testing

CYP2D6 genetic test

21. Have you ever ordered a *CYP2D6* genetic test?

- ◯ Yes
- ◯ No

22. If you have ordered this *CYP2D6* genetic test, did you feel confident with interpreting the results to inform prescribing?

- Yes
- No
- The testing facility interpreted the results for me

23. Do you have local (institutional or national) guidance to action *CYP2D6* results?

- Yes
- No
- Unsure

24. Have you ever been presented with *CYP2D6* genetic testing results that you did not request from a patient you are treating?

- Yes
- No

Prescribing choices

25. If you have been presented with this *CYP2D6* genetic information by a patient, did the information change your prescribing choices for this patient?

- Yes
- No

26. Would you want to know your patients' *CYP2D6* genetic testing results before prescribing a medicine metabolised by CYP2D6?

- Yes
- No
- I would only want this information if the turnaround time matched my requirements
- Unsure

27. Do you have access to institutionally, nationally, or internationally standardised written patient education material for patients regarding *CYP2D6* testing?

- Yes
- No
- Unsure

Drug-Drug interactions

28. Do you think that the magnitude of drug-drug interactions can be modified by the *CYP2C19* or *CYP2D6* genotype?

- Yes
- No
- Unsure

29. Do you think that the magnitude of drug-drug interactions can be modified by a medical comorbidity like obesity, diabetes, liver disease or renal impairment?

- ◯ Yes
- ◯ No
- ◯ Unsure

Health Equality

30. Do you think that *CYP2C19* or *CYP2D6* genotyping would...?

- ◯ Have no impact on health equality
- ◯ Worsen health equality
- ◯ Improve health equality

31. Do you think that ancestry impacts on *CYP2C19* or *CYP2D6* genotypes?

- ◯ Yes
- ◯ No
- ◯ Unsure

Personal experience

32. Have you ever taken a medicine metabolised by CYP2C19 yourself?

- ◯ Yes
- ◯ No
- ◯ Unsure

33. Have you ever taken a medicine metabolised by CYP2D6 yourself?

- ◯ Yes
- ◯ No
- ◯ Unsure

34. Have you personally chosen to undertake *CYP2C19* genetic testing?

- ◯ Yes
- ◯ No

35. Have you personally chosen to undertake *CYP2D6* genetic testing?

- ◯ Yes
- ◯ No

36. Would you like to be offered *CYP2C19* genetic testing if you had an indication to receive a medication metabolised by CYP2C19?

- ◯ Yes
- ◯ No
- ◯ Unsure

37. Would you like to be offered *CYP2D6* genetic testing if you had an indication to receive a medication metabolised by CYP2D6?

- Yes
- No
- Unsure

38. Would you pay for a private *CYP2C19* genetic test?

- Yes
- No
- Unsure

39. Would you pay for a private *CYP2D6* genetic test?

- Yes
- No
- Unsure

If willing to pay for a private test

40. If you would pay for a private test, how much would you pay, in Euros (maximum)?

Demographics

41. What is your professional role?

- Physician – General Cardiology
- Physician – Sub-specialty in cardiology (heart failure, arrythmia, imaging, acute, prevention)
- Physician – Internal Medicine
- Physician – General Practice
- Other (please specify)
- Nurse / Advanced Nurse Practitioner
- Scientist / researcher
- Other physician
- Other allied professional

42. What is your age?

- Younger than 30
- 30-39
- 40-49
- 50-59
- 60-69
- 70 or older

43. What is your gender?

- Female
- Male
- Other
- Do not want to say

44. Which country do you work in?

45. Which race or ethnicity best describes you? Please choose only one.

- American Indian or Alaskan Native
- Asian / Pacific Islander
- Black or African American
- Hispanic
- White / Caucasian
- Multiple ethnicity
- Other (please specify)
- Prefer not to answer
