# Supplementary material for: Cardiovascular prescriber attitudes to pharmacogenomics: a survey by the ESC working group on cardiovascular pharmacotherapy
Source: Pharmacogenomics J. 2026 Apr 23;26(3):17. doi: 10.1038/s41397-026-00412-6 (PMC13106032; doi:10.1038/s41397-026-00412-6)
Supplement: Supplementary file 2 — Supplement 2 [file 41397_2026_412_MOESM2_ESM.pdf]

Q2 Do you prescribe medicines?

Answered: 290 Skipped: 0

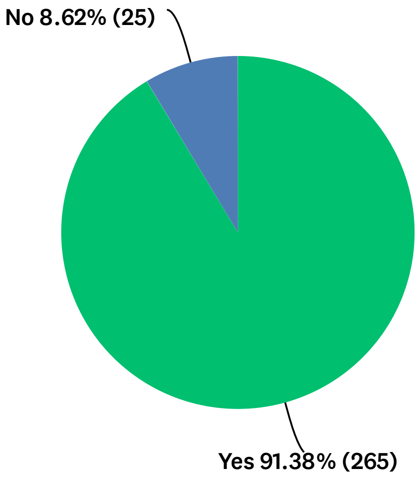

Q3 How adherent do you think your patients are generally with medication

Answered: 205 Skipped: 85

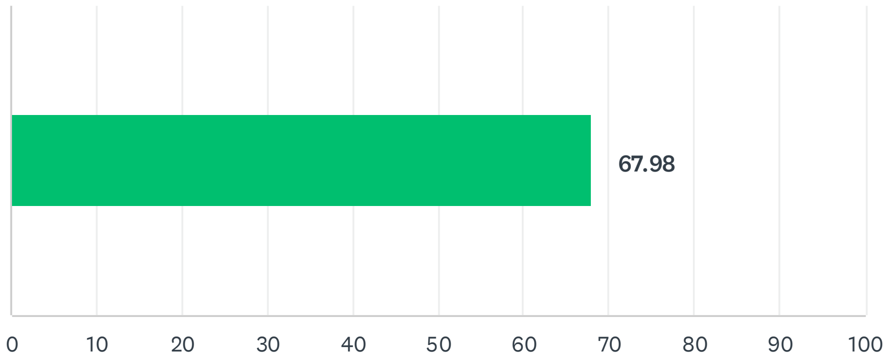

#		DATE
1	94	8/25/2024 7:45 PM
2	92	8/2/2024 9:11 AM
3	46	6/12/2024 1:12 PM
4	50	6/10/2024 7:14 AM
5	70	5/31/2024 7:41 AM
6	70	5/24/2024 7:10 AM
7	80	5/21/2024 8:34 PM
8	40	5/21/2024 12:25 AM
9	50	5/20/2024 10:47 PM
10	90	5/19/2024 11:42 AM
11	65	5/18/2024 10:51 PM
12	46	5/18/2024 12:59 PM
13	87	5/17/2024 11:54 PM
14	60	5/17/2024 10:47 AM
15	70	5/15/2024 4:13 PM
16	72	5/15/2024 1:10 PM
17	85	5/15/2024 10:55 AM
18	50	5/14/2024 6:43 PM
19	20	5/14/2024 2:11 PM
20	1	5/13/2024 1:20 PM
21	75	5/13/2024 6:07 AM
22	50	5/12/2024 10:15 AM
23	74	5/10/2024 8:49 PM

24	68	5/10/2024 2:28 PM
25	92	5/9/2024 8:59 PM
26	91	5/9/2024 5:32 PM
27	70	5/9/2024 4:54 PM
28	66	5/9/2024 9:22 AM
29	74	5/9/2024 7:46 AM
30	83	5/9/2024 3:11 AM
31	90	5/9/2024 2:50 AM
32	60	5/9/2024 2:05 AM
33	50	5/8/2024 10:42 PM
34	80	5/8/2024 10:02 PM
35	70	5/8/2024 8:49 PM
36	50	5/8/2024 7:21 PM
37	60	5/8/2024 2:50 PM
38	75	5/8/2024 1:36 PM
39	71	5/8/2024 10:27 AM
40	75	5/8/2024 4:05 AM
41	70	5/8/2024 1:59 AM
42	79	5/8/2024 1:19 AM
43	61	5/8/2024 12:54 AM
44	50	5/7/2024 9:02 PM
45	40	5/7/2024 6:44 PM
46	52	5/7/2024 6:20 PM
47	63	5/7/2024 5:32 PM
48	50	5/7/2024 4:03 PM
49	75	5/7/2024 2:40 PM
50	63	5/7/2024 2:19 PM
51	56	5/7/2024 12:45 PM
52	46	5/7/2024 12:37 PM
53	71	5/7/2024 11:18 AM
54	85	5/7/2024 11:18 AM
55	69	5/7/2024 10:48 AM
56	80	5/7/2024 10:18 AM
57	50	5/7/2024 10:17 AM
58	62	5/7/2024 10:16 AM
59	70	5/7/2024 9:12 AM
60	40	5/7/2024 8:56 AM
61	70	5/7/2024 8:44 AM

62	81	5/7/2024 8:06 AM
63	86	5/7/2024 7:38 AM
64	70	5/7/2024 7:38 AM
65	65	5/7/2024 7:21 AM
66	75	5/7/2024 5:07 AM
67	80	5/7/2024 4:12 AM
68	80	5/7/2024 3:27 AM
69	65	5/7/2024 2:47 AM
70	80	5/7/2024 12:58 AM
71	71	5/6/2024 11:56 PM
72	90	5/6/2024 10:57 PM
73	80	5/6/2024 10:56 PM
74	35	5/6/2024 10:52 PM
75	80	5/6/2024 10:30 PM
76	71	5/6/2024 9:46 PM
77	55	5/6/2024 9:36 PM
78	71	5/6/2024 8:55 PM
79	90	5/6/2024 8:55 PM
80	76	5/6/2024 8:16 PM
81	80	5/6/2024 8:08 PM
82	30	5/6/2024 7:15 PM
83	44	5/6/2024 7:08 PM
84	58	5/6/2024 6:56 PM
85	90	5/6/2024 6:52 PM
86	75	5/6/2024 6:45 PM
87	41	5/6/2024 6:44 PM
88	99	5/6/2024 6:38 PM
89	71	5/6/2024 6:22 PM
90	41	5/6/2024 6:17 PM
91	80	5/6/2024 5:54 PM
92	77	5/6/2024 5:52 PM
93	60	5/6/2024 5:47 PM
94	50	5/6/2024 5:46 PM
95	50	5/6/2024 5:41 PM
96	86	5/6/2024 5:40 PM
97	70	5/6/2024 5:34 PM
98	53	5/6/2024 4:46 PM
99	72	5/6/2024 4:19 PM

100	75	5/6/2024 4:09 PM
101	76	5/6/2024 4:08 PM
102	85	5/6/2024 4:05 PM
103	81	5/6/2024 3:38 PM
104	66	5/6/2024 3:38 PM
105	20	5/6/2024 3:36 PM
106	95	5/6/2024 3:35 PM
107	91	5/6/2024 3:27 PM
108	60	5/6/2024 3:19 PM
109	75	5/6/2024 3:18 PM
110	75	5/6/2024 3:16 PM
111	70	5/6/2024 3:05 PM
112	66	5/6/2024 2:59 PM
113	73	5/6/2024 2:52 PM
114	68	5/6/2024 2:45 PM
115	50	5/6/2024 2:43 PM
116	47	5/6/2024 2:40 PM
117	66	5/6/2024 2:23 PM
118	80	5/6/2024 1:56 PM
119	81	5/6/2024 1:54 PM
120	65	5/6/2024 1:52 PM
121	100	5/6/2024 1:49 PM
122	80	5/6/2024 1:45 PM
123	60	5/6/2024 1:38 PM
124	80	5/6/2024 1:35 PM
125	60	5/6/2024 1:19 PM
126	1	5/6/2024 1:18 PM
127	44	5/6/2024 1:14 PM
128	75	5/6/2024 1:08 PM
129	50	5/6/2024 1:08 PM
130	80	5/6/2024 1:03 PM
131	92	5/6/2024 1:03 PM
132	70	5/6/2024 12:56 PM
133	62	5/6/2024 12:31 PM
134	65	5/6/2024 12:29 PM
135	80	5/6/2024 12:29 PM
136	89	5/6/2024 12:20 PM
137	72	5/6/2024 12:17 PM

138	70	5/6/2024 12:12 PM
139	70	5/6/2024 12:12 PM
140	70	5/6/2024 12:01 PM
141	73	5/6/2024 11:59 AM
142	43	5/6/2024 11:56 AM
143	59	5/6/2024 11:55 AM
144	70	5/6/2024 11:50 AM
145	80	5/6/2024 11:50 AM
146	54	5/6/2024 11:42 AM
147	71	5/6/2024 11:40 AM
148	87	5/6/2024 11:38 AM
149	85	5/6/2024 11:37 AM
150	69	5/6/2024 11:36 AM
151	75	5/6/2024 11:34 AM
152	61	5/6/2024 11:31 AM
153	66	5/6/2024 11:30 AM
154	70	5/6/2024 11:29 AM
155	50	5/6/2024 11:29 AM
156	81	5/6/2024 11:21 AM
157	100	5/6/2024 11:20 AM
158	75	5/6/2024 11:14 AM
159	60	5/6/2024 11:14 AM
160	60	5/6/2024 11:08 AM
161	60	5/6/2024 11:05 AM
162	50	5/6/2024 11:01 AM
163	52	5/6/2024 11:00 AM
164	80	5/6/2024 10:49 AM
165	70	5/6/2024 10:48 AM
166	80	5/6/2024 10:47 AM
167	76	5/6/2024 10:46 AM
168	50	5/6/2024 10:44 AM
169	75	5/6/2024 10:43 AM
170	96	5/6/2024 10:40 AM
171	82	5/6/2024 10:31 AM
172	80	5/6/2024 10:31 AM
173	90	5/6/2024 10:31 AM
174	75	5/6/2024 10:30 AM
175	80	5/6/2024 10:29 AM

176	19	5/6/2024 10:28 AM
177	53	5/6/2024 10:28 AM
178	51	5/6/2024 10:27 AM
179	90	5/6/2024 10:27 AM
180	80	5/6/2024 10:26 AM
181	68	5/6/2024 10:22 AM
182	85	5/6/2024 10:22 AM
183	70	5/6/2024 10:21 AM
184	71	5/6/2024 10:21 AM
185	64	5/6/2024 10:20 AM
186	62	5/6/2024 10:20 AM
187	75	5/6/2024 10:20 AM
188	85	5/6/2024 10:19 AM
189	80	5/6/2024 10:18 AM
190	75	5/6/2024 10:18 AM
191	60	5/6/2024 10:18 AM
192	54	5/6/2024 10:16 AM
193	55	5/6/2024 10:14 AM
194	70	5/6/2024 10:10 AM
195	100	5/6/2024 10:09 AM
196	59	5/6/2024 10:09 AM
197	70	5/6/2024 10:08 AM
198	85	5/6/2024 10:07 AM
199	55	5/6/2024 10:07 AM
200	60	5/6/2024 10:07 AM
201	80	5/6/2024 10:05 AM
202	67	5/6/2024 10:04 AM
203	80	5/6/2024 10:04 AM
204	60	5/6/2024 10:03 AM
205	40	4/29/2024 7:50 PM

Q4 How often do you prescribe medicine metabolised by the Cytochrome P450 2C19 (CYP2C19) enzyme (examples include clopidogrel, mavacamten, proton pump inhibitors, tricyclic antidepressants, citalopram, sertraline)?

Answered: 238 Skipped: 52

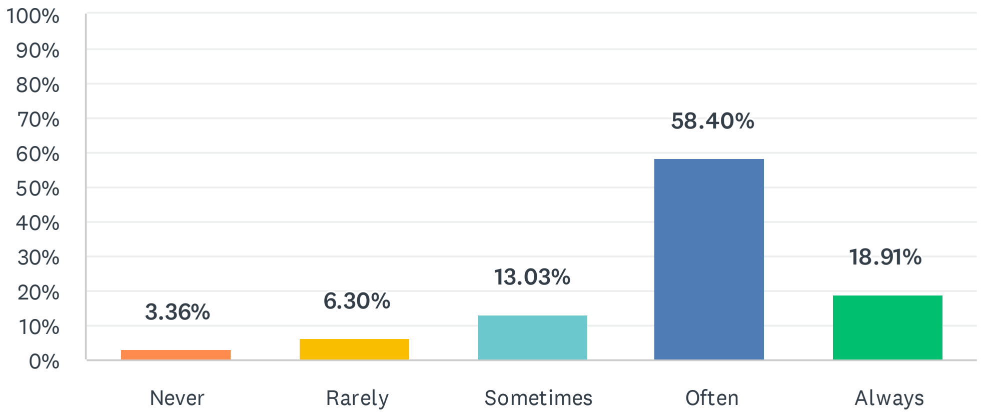

Q5 Do you think that variability in response to these medicines metabolised by CYP2C19 causes significant problems for your patients?

Answered: 238 Skipped: 52

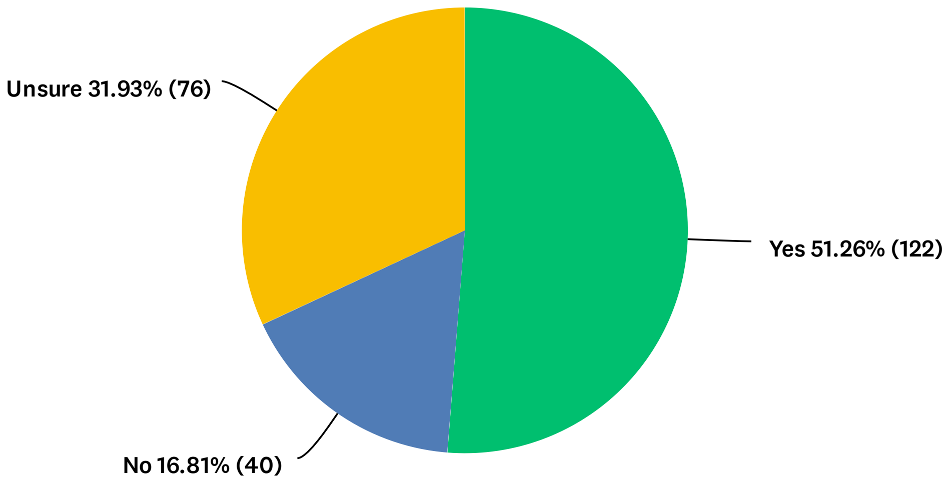

Q6 Do you think that testing the CYP2C19 gene, which encodes the CYP2C19 enzyme, could improve the risk benefit ratio of at least one CYP2C19 metabolised medicine for your patients?

Answered: 236 Skipped: 54

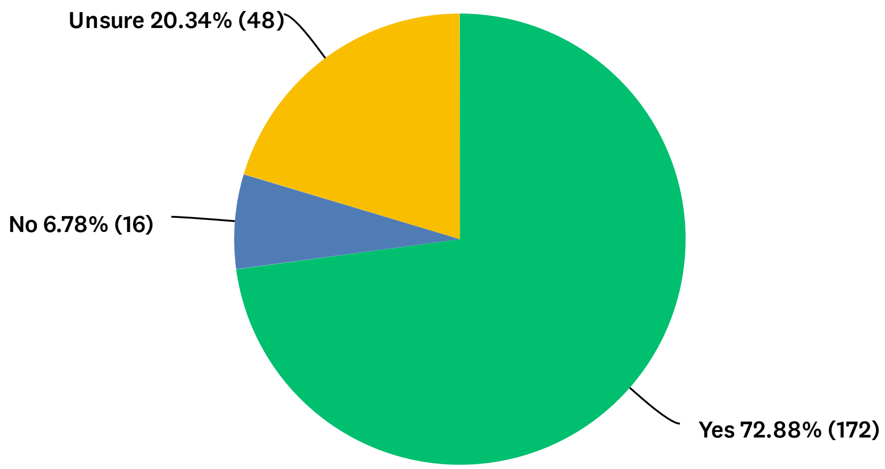

Q7 Do you think testing the CYP2C19 gene could improve medication adherence?

Answered: 237 Skipped: 53

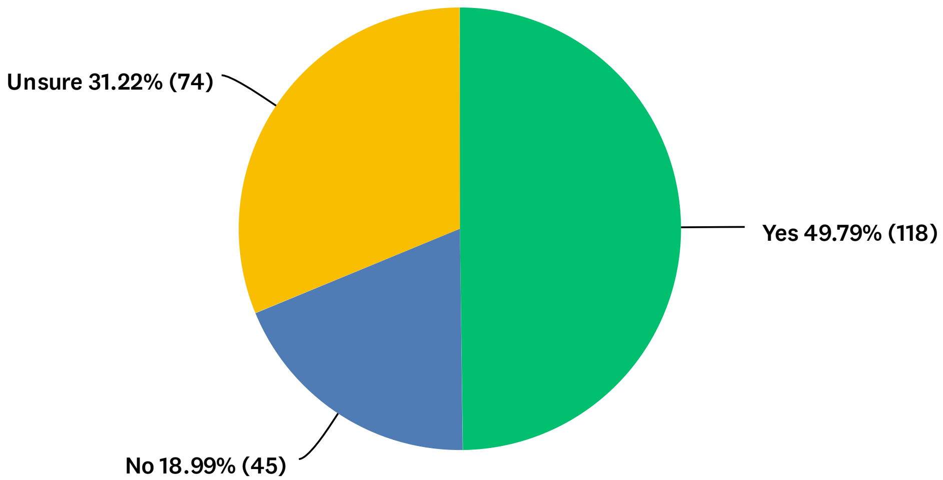

Q8 Do you have access to CYP2C19 genetic testing? Tick all that apply

Answered: 237 Skipped: 53

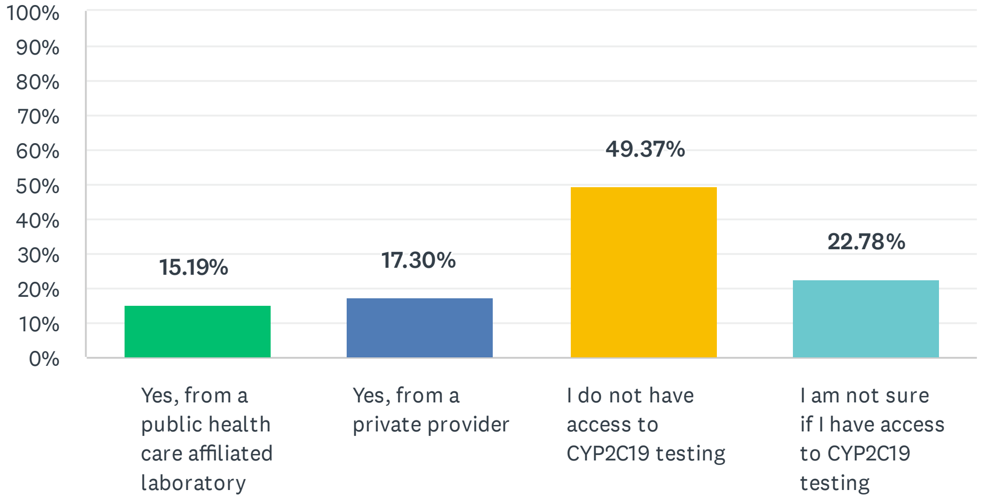

Q9 Have you ever ordered a CYP2C19 genetic test?

Answered: 72   Skipped: 218

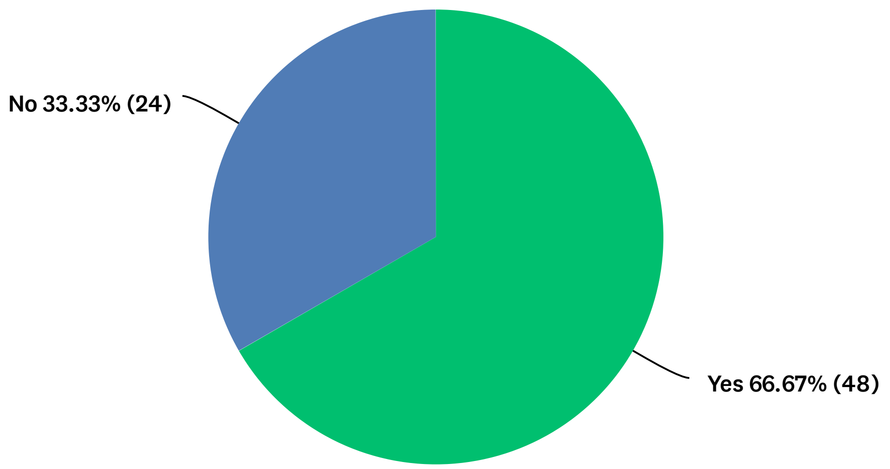

Q10 If you have ordered this CYP2C19 genetic test, did you feel confident with interpreting the results to inform prescribing?

Answered: 65 Skipped: 225

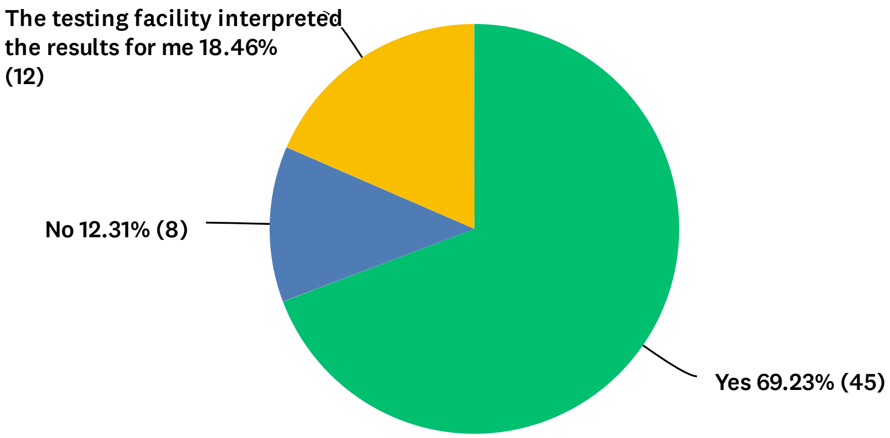

Q11 Do you have local (institutional or national) guidance to action CYP2C19 results?

Answered: 234 Skipped: 56

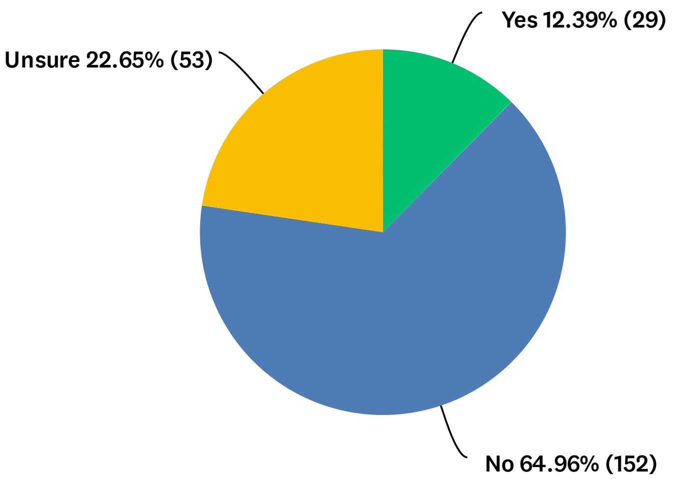

Q12 Have you ever been presented with CYP2C19 genetic testing results that you did not request from a patient you are treating?

Answered: 234 Skipped: 56

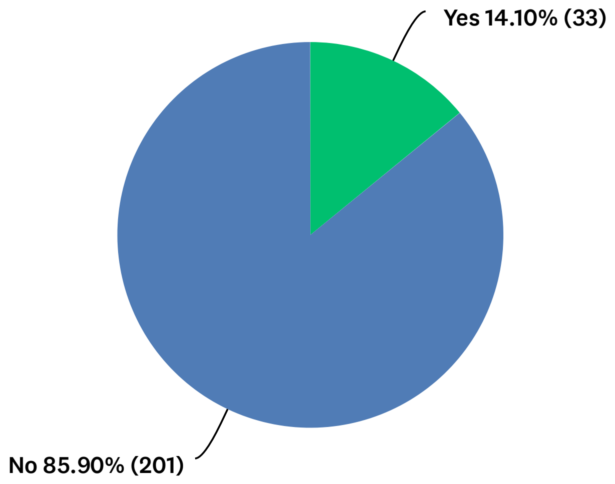

Q13 If you have been presented with CYP2C19 genetic testing information by a patient, did the genetic information presented change your prescribing choices for this patient?

Answered: 34 Skipped: 256

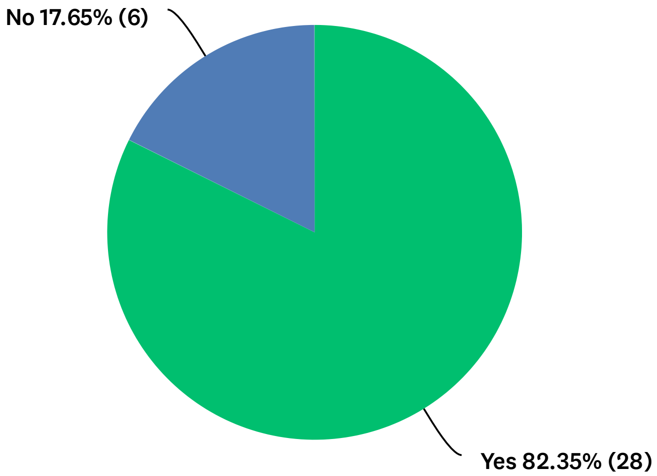

Q14 Would you want to know your patients' CYP2C19 genetic testing results before prescribing a medicine metabolised by CYP2C19?

Answered: 221 Skipped: 69

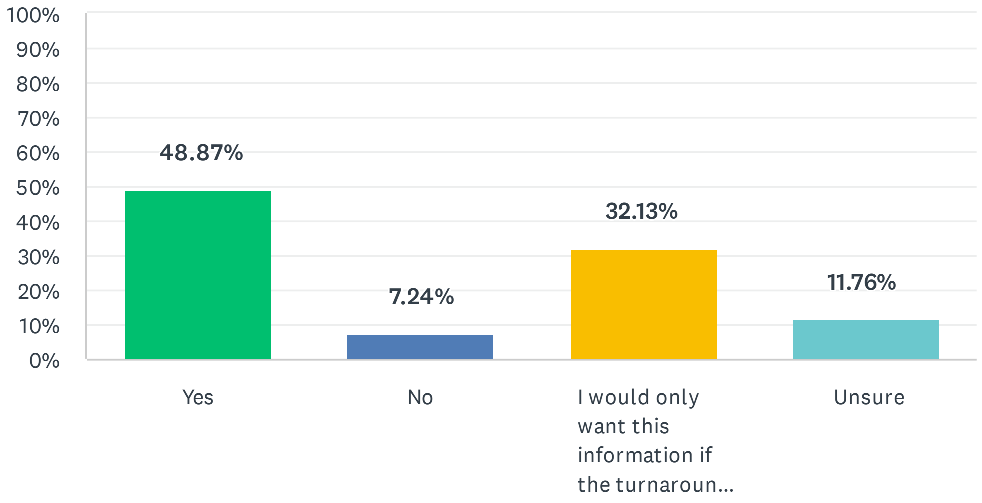

Q15 Do you have access to institutionally, nationally, or internationally standardised written patient education material for patients regarding CYP2C19 testing?

Answered: 218 Skipped: 72

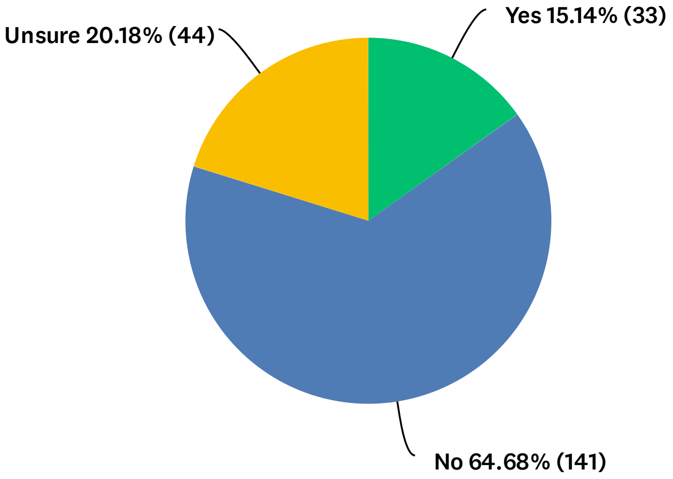

Q16 How often do you prescribe medicine metabolised by the Cytochrome P450 2D6 (CYP2D6) enzyme (examples include metoprolol, flecainide, ondansetron, codeine, tramadol, tamoxifen, paroxetine, some tricyclic antidepressants, many antipsychotics)?

Answered: 220 Skipped: 70

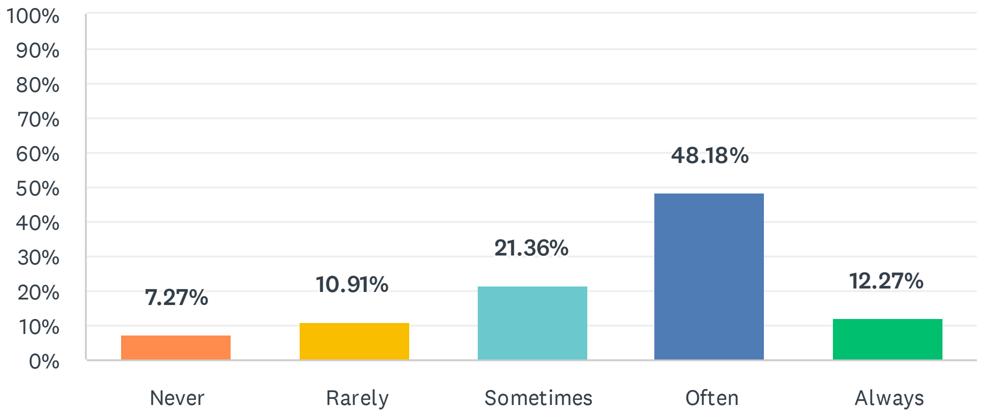

Q17 Do you think that variability in response to these medicines metabolised by CYP2D6 causes significant problems for your patients?

Answered: 219 Skipped: 71

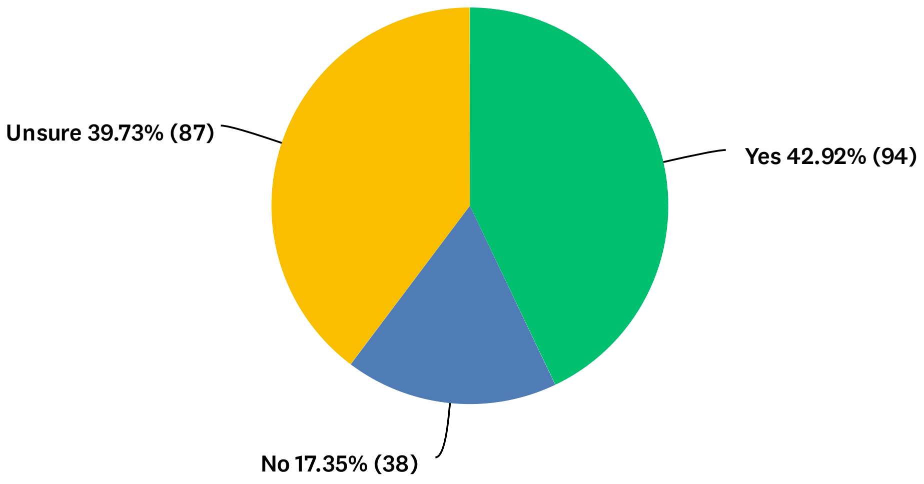

Q18 Do you think that testing the CYP2D6 gene, which encodes the CYP2D6 enzyme, could improve the risk benefit ratio of at least one CYP2D6 metabolised medicine for your patients?

Answered: 220 Skipped: 70

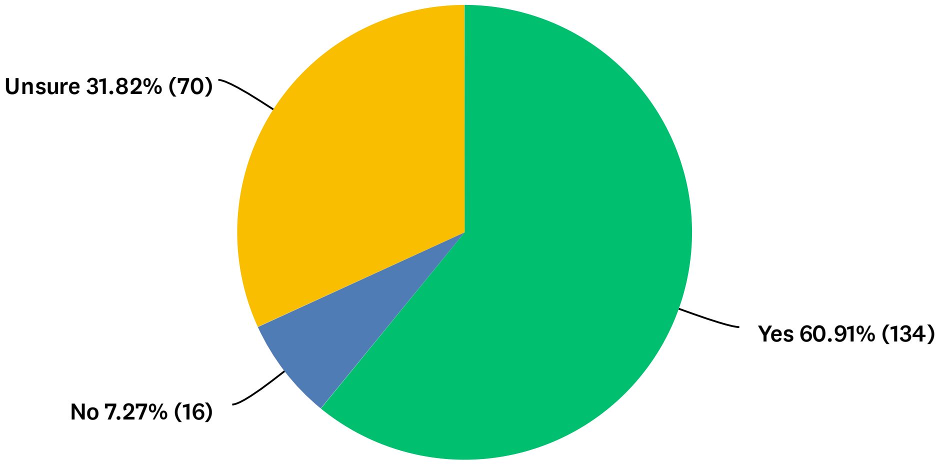

Q19 Do you think testing the CYP2D6 gene could improve medication adherence?

Answered: 220 Skipped: 70

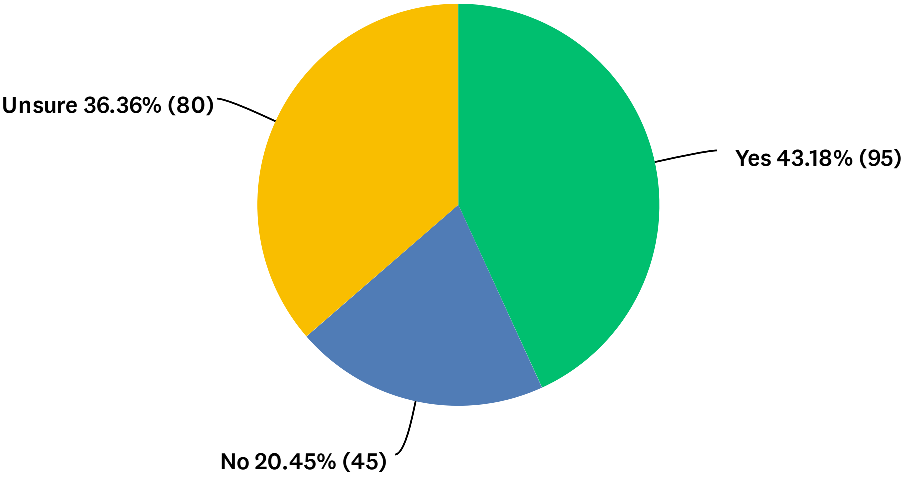

Q20 Do you have access to CYP2D6 genetic testing? Tick all that apply

Answered: 219 Skipped: 71

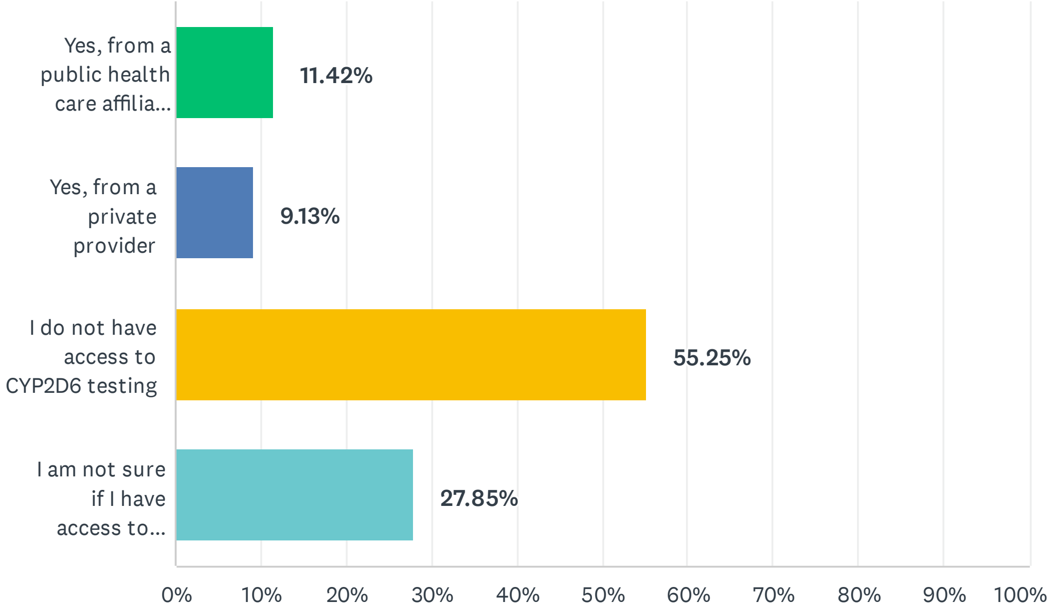

Q21 Have you ever ordered a CYP2D6 genetic test?

Answered: 42 Skipped: 248

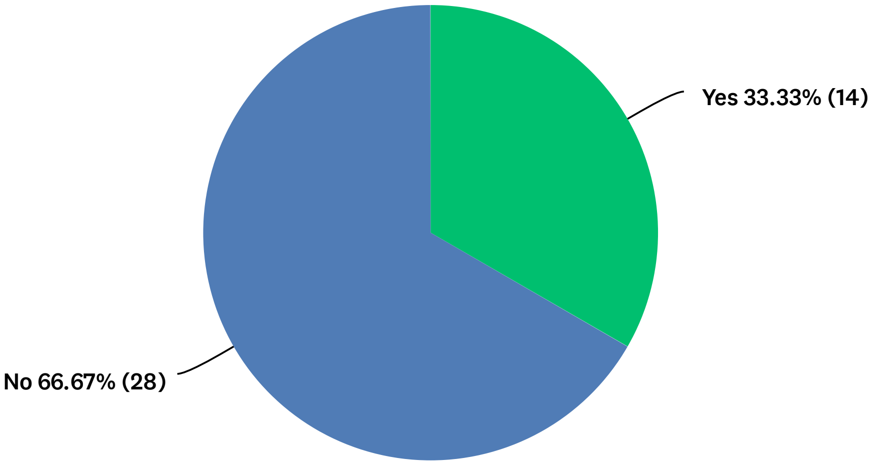

Q22 If you have ordered this CYP2D6 genetic test, did you feel confident with interpreting the results to inform prescribing?

Answered: 34 Skipped: 256

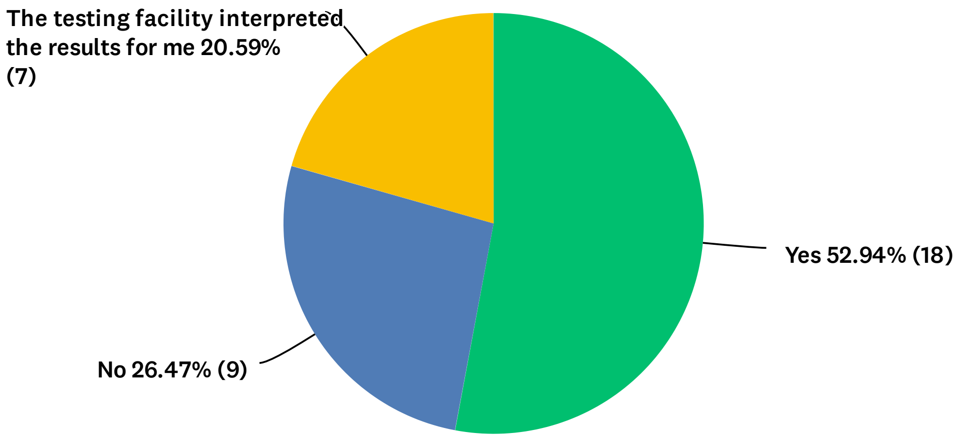

Q23 Do you have local (institutional or national) guidance to action CYP2D6 results?

Answered: 203 Skipped: 87

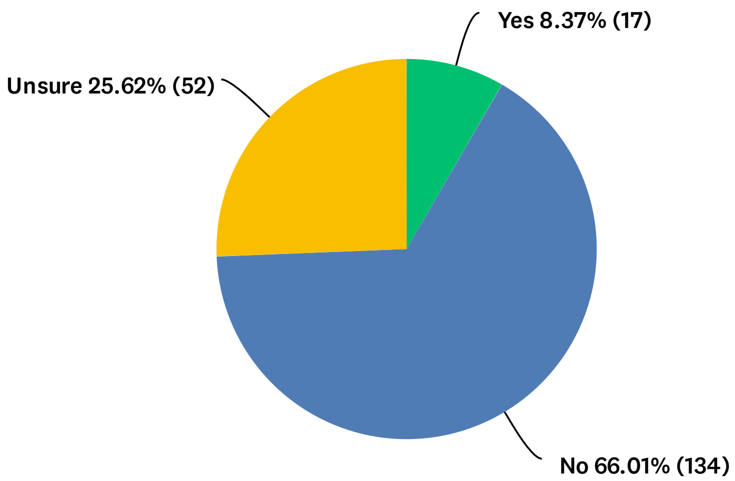

Q24 Have you ever been presented with CYP2D6 genetic testing results that you did not request from a patient you are treating?

Answered: 203 Skipped: 87

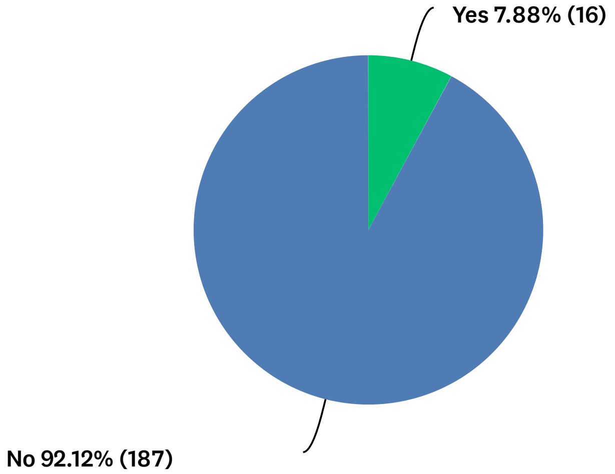

Q25 If you have been presented with this CYP2D6 genetic information by a patient, did the information change your prescribing choices for this patient?

Answered: 32 Skipped: 258

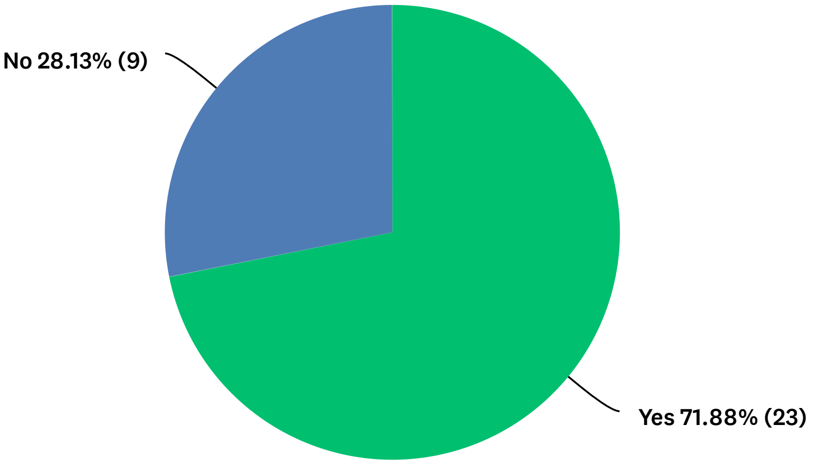

Q26 Would you want to know your patients' CYP2D6 genetic testing results before prescribing a medicine metabolised by CYP2D6?

Answered: 211 Skipped: 79

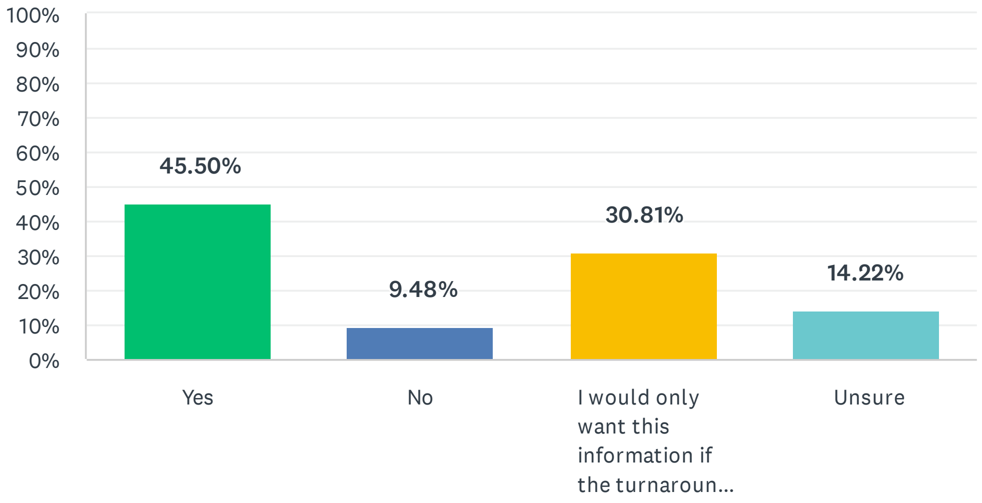

Q27 Do you have access to institutionally, nationally, or internationally standardised written patient education material for patients regarding CYP2D6 testing?

Answered: 208 Skipped: 82

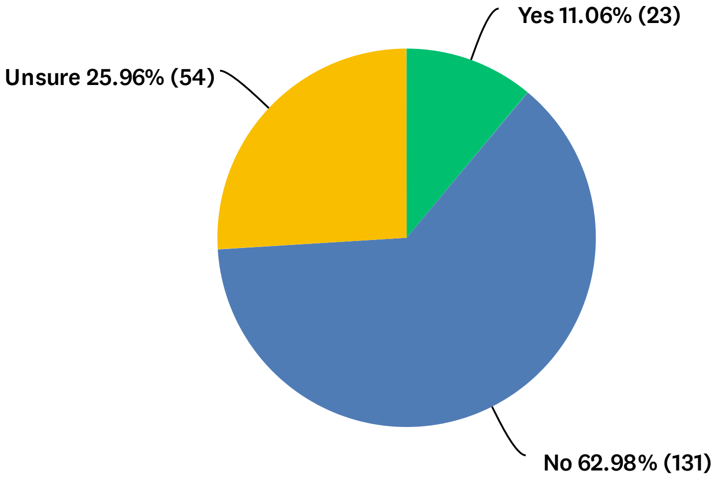

Q28 Do you think that the magnitude of drug-drug interactions can be modified by the CYP2C19 or CYP2D6 genotype?

Answered: 211 Skipped: 79

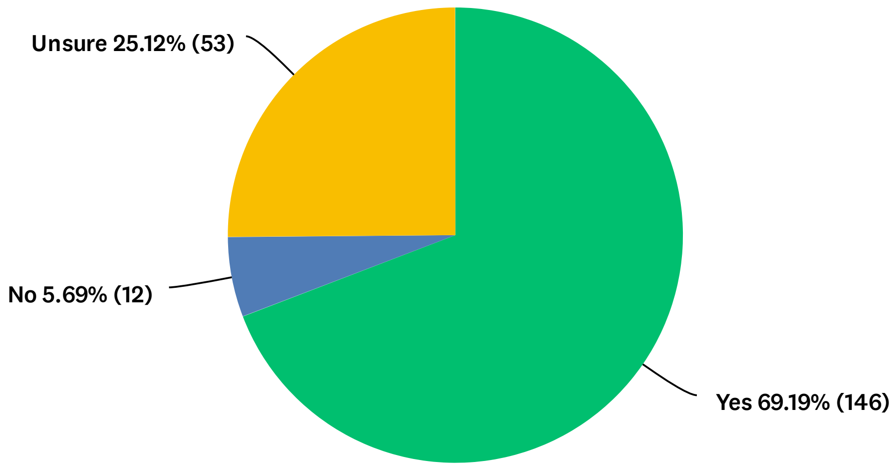

Q29 Do you think that the magnitude of drug-drug interactions can be modified by a medical comorbidity like obesity, diabetes, liver disease or renal impairment?

Answered: 210 Skipped: 80

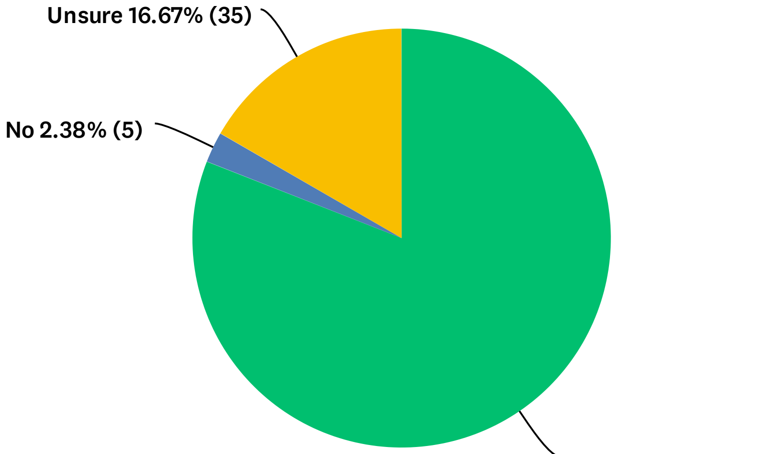

Q30 Do you think that CYP2C19 or CYP2D6 genotyping would...?

Answered: 209 Skipped: 81

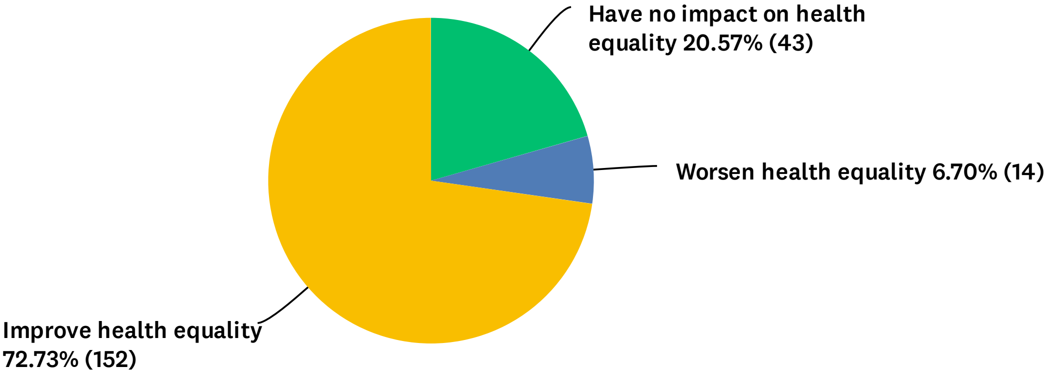

Q31 Do you think that ancestry impacts on CYP2C19 or CYP2D6 genotypes?

Answered: 206 Skipped: 84

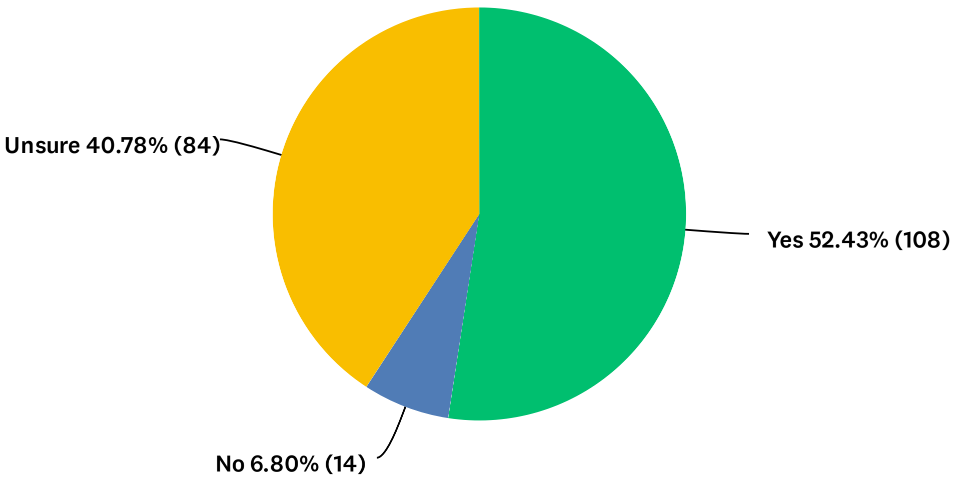

Q32 Have you ever taken a medicine metabolised by CYP2C19 yourself?

Answered: 210 Skipped: 80

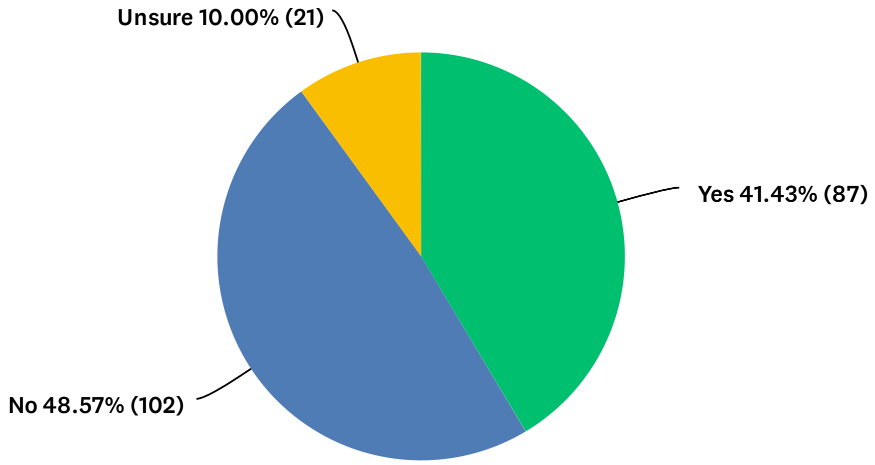

Q33 Have you ever taken a medicine metabolised by CYP2D6 yourself?

Answered: 210 Skipped: 80

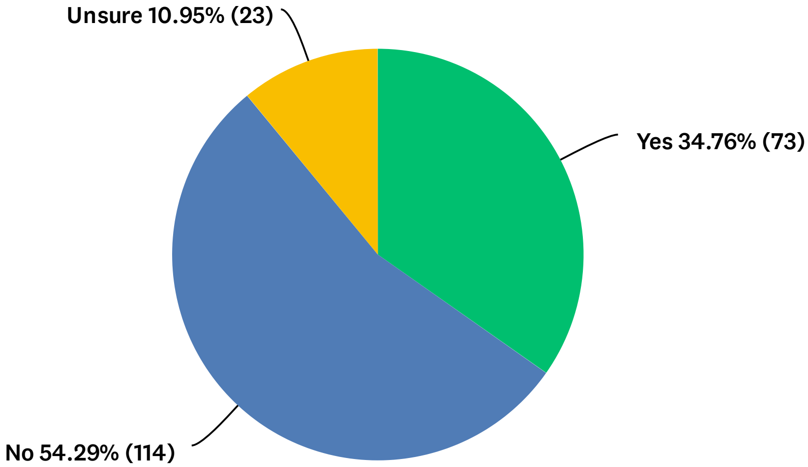

Q34 Have you personally chosen to undertake CYP2C19 genetic testing?

Answered: 209 Skipped: 81

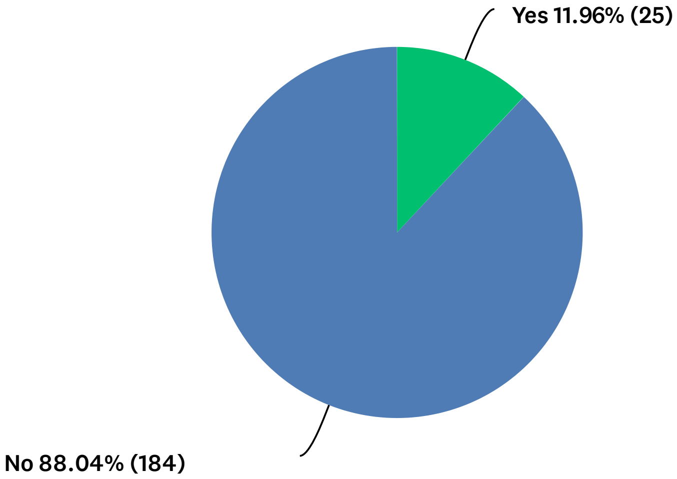

Q35 Have you personally chosen to undertake CYP2D6 genetic testing?

Answered: 209   Skipped: 81

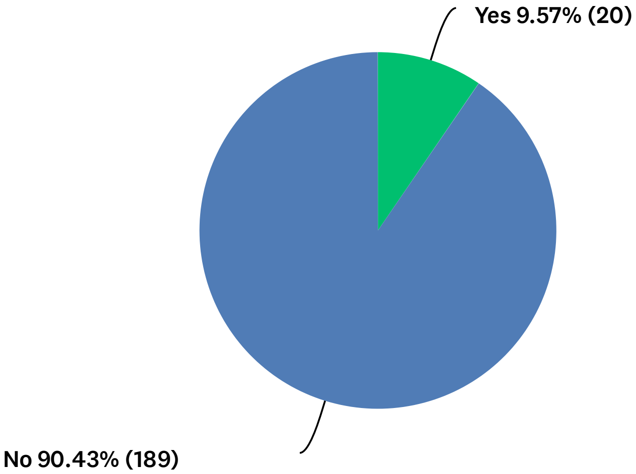

Q36 Would you like to be offered CYP2C19 genetic testing if you had an indication to receive a medication metabolised by CYP2C19?

Answered: 210 Skipped: 80

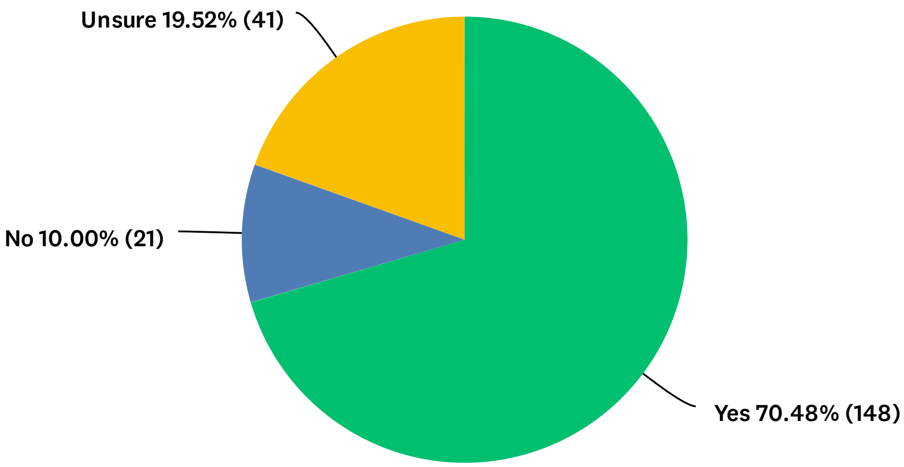

Q37 Would you like to be offered CYP2D6 genetic testing if you had an indication to receive a medication metabolised by CYP2D6?

Answered: 210 Skipped: 80

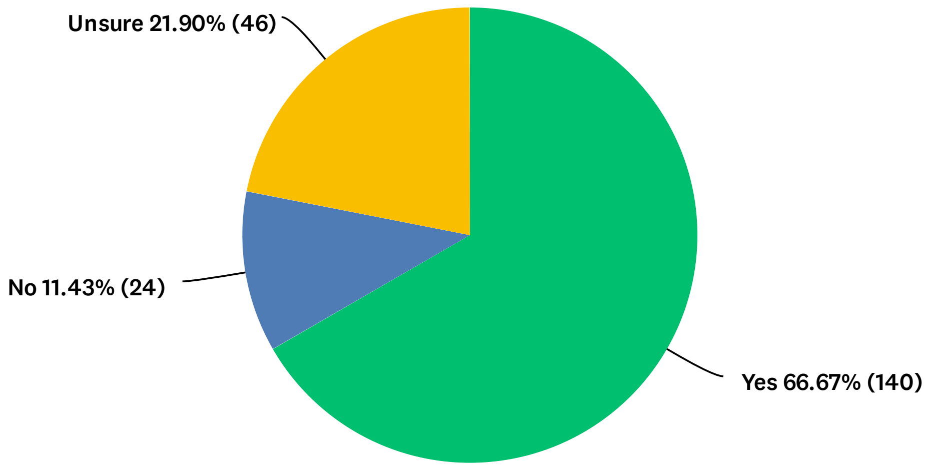

Q38 Would you pay for a private CYP2C19 genetic test?

Answered: 210 Skipped: 80

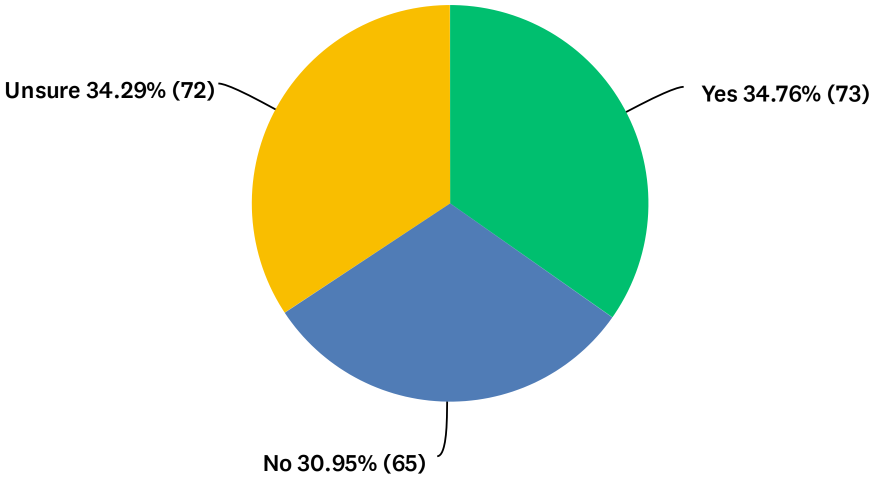

Q39 Would you pay for a private CYP2D6 genetic test?

Answered: 209 Skipped: 81

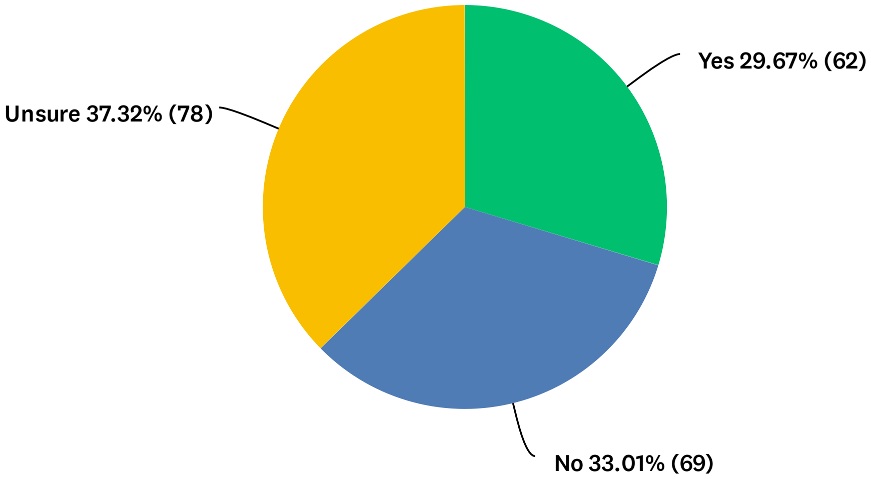

Q40 If you would pay for a private test, how much would you pay, in Euros (maximum)?

Answered: 68 Skipped: 222

#	RESPONSES	DATE
1	25	6/12/2024 1:15 PM
2	80 Euro	5/24/2024 7:18 AM
3	50	5/21/2024 8:38 PM
4	30	5/19/2024 11:47 AM
5	200	5/18/2024 12:03 AM
6	250	5/15/2024 1:18 PM
7	50	5/13/2024 6:09 AM
8	300.00	5/12/2024 10:22 AM
9	I do not have a price reference for the studies	5/9/2024 5:05 PM
10	80	5/9/2024 2:07 AM
11	2	5/8/2024 1:39 PM
12	20	5/8/2024 10:30 AM
13	50	5/8/2024 2:04 AM
14	2000	5/7/2024 6:22 PM
15	25	5/7/2024 12:47 PM
16	0	5/7/2024 12:40 PM
17	Not sure	5/7/2024 11:23 AM
18	40	5/7/2024 10:53 AM
19	20	5/7/2024 10:20 AM
20	100	5/7/2024 9:04 AM
21	100	5/7/2024 7:43 AM
22	100	5/7/2024 5:15 AM
23	200	5/7/2024 4:18 AM
24	80	5/7/2024 1:03 AM
25	150	5/7/2024 12:01 AM
26	100 euros	5/6/2024 11:08 PM
27	150	5/6/2024 10:54 PM
28	25	5/6/2024 8:22 PM
29	100	5/6/2024 7:24 PM
30	100	5/6/2024 6:49 PM
31	500	5/6/2024 5:56 PM

32	100	5/6/2024 4:48 PM
33	100	5/6/2024 4:23 PM
34	100	5/6/2024 4:15 PM
35	100	5/6/2024 3:40 PM
36	No	5/6/2024 3:38 PM
37	50	5/6/2024 3:19 PM
38	200	5/6/2024 2:54 PM
39	50	5/6/2024 2:41 PM
40	30	5/6/2024 2:40 PM
41	200	5/6/2024 2:34 PM
42	20	5/6/2024 1:58 PM
43	50	5/6/2024 1:11 PM
44	30	5/6/2024 12:36 PM
45	100	5/6/2024 12:14 PM
46	50	5/6/2024 12:07 PM
47	50	5/6/2024 11:58 AM
48	100	5/6/2024 11:41 AM
49	100	5/6/2024 11:40 AM
50	100	5/6/2024 11:34 AM
51	300	5/6/2024 11:33 AM
52	150	5/6/2024 11:26 AM
53	NA	5/6/2024 11:21 AM
54	25	5/6/2024 11:17 AM
55	50	5/6/2024 11:10 AM
56	50	5/6/2024 11:07 AM
57	50€	5/6/2024 10:54 AM
58	100	5/6/2024 10:53 AM
59	50	5/6/2024 10:50 AM
60	25	5/6/2024 10:34 AM
61	75	5/6/2024 10:32 AM
62	25	5/6/2024 10:31 AM
63	25	5/6/2024 10:29 AM
64	70	5/6/2024 10:24 AM
65	20	5/6/2024 10:24 AM
66	50	5/6/2024 10:12 AM
67	50	4/29/2024 8:45 PM
68	100	4/29/2024 7:57 PM

Q41 What is your professional role?

Answered: 209   Skipped: 81

ANSWER CHOICES	RESPONSES	
Physician – General Cardiology	40.67%	85
Physician – Sub-specialty in cardiology (heart failure, arrythmia, imaging, acute, prevention)	30.14%	63
Physician – Internal Medicine	11.48%	24
Other (please specify)	6.22%	13
Physician – General Practice	4.78%	10
Other physician	3.83%	8
Scientist / researcher	1.91%	4
Nurse / Advanced Nurse Practitioner	0.96%	2
TOTAL		209

#	OTHER (PLEASE SPECIFY)	DATE
1	int card	5/8/2024 10:05 PM
2	Physician - Diabetologist	5/7/2024 2:44 PM
3	Interventional Cardiology	5/7/2024 8:47 AM
4	uci coordinator	5/7/2024 1:58 AM
5	gp with genetic specislity	5/7/2024 1:04 AM
6	Internal Medicine, Oncology	5/6/2024 5:57 PM
7	Physician-Sub-specialty in cardiology (heart failure and imaging) currently retired	5/6/2024 2:07 PM
8	Medical oncologist	5/6/2024 12:22 PM
9	Urgent and functional cardiologist (ECG, Holter, Veloergometry, Treadmill, EchoCG).	5/6/2024 11:29 AM
10	pulmonology	5/6/2024 10:25 AM
11	Neurology	5/6/2024 10:23 AM
12	Physician - Inmunology	5/6/2024 10:14 AM
13	Cardiology/ Interventional	4/29/2024 7:59 PM

Q42 What is your age?

Answered: 207 Skipped: 83

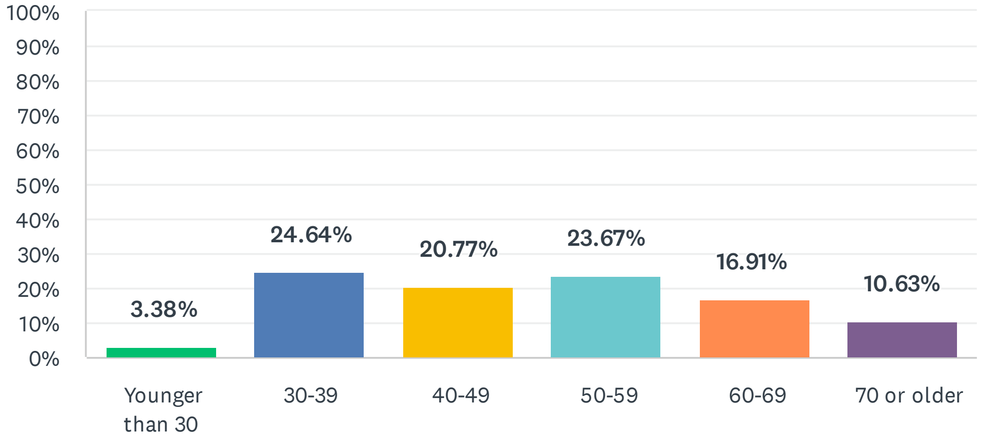

Q43 What is your gender?

Answered: 206 Skipped: 84

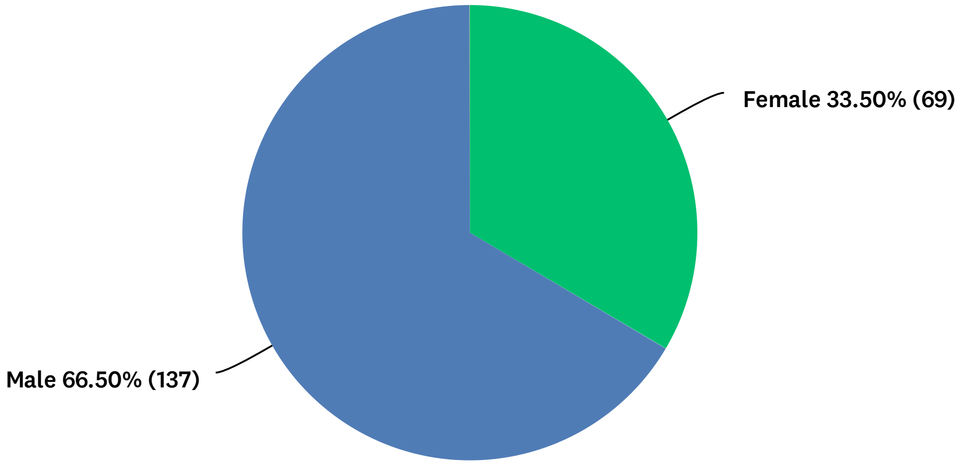

Q44 Which country do you work in?

Answered: 202 Skipped: 88

ANSWER CHOICES	RESPONSES	
Greece	5.94%	12
Italy	5.45%	11
United Kingdom of Great Britain and Northern Ireland	5.45%	11
Spain	4.46%	9
Romania	3.96%	8
India	3.47%	7
Japan	3.47%	7
Argentina	2.97%	6
Netherlands (The)	2.97%	6
Poland	2.97%	6
Ukraine	2.97%	6
Croatia	2.48%	5
Czechia	2.48%	5
Germany	2.48%	5
United States of America	2.48%	5
Brazil	1.98%	4
Mexico	1.98%	4
Serbia	1.98%	4
Sweden	1.98%	4
Australia	1.49%	3
Denmark	1.49%	3
Egypt	1.49%	3
Indonesia	1.49%	3
Latvia	1.49%	3
New Zealand	1.49%	3
Armenia	0.99%	2
Austria	0.99%	2
Bulgaria	0.99%	2
Canada	0.99%	2
France	0.99%	2
Georgia	0.99%	2
Hungary	0.99%	2

Morocco	0.99%	2
Pakistan	0.99%	2
Philippines	0.99%	2
Slovakia	0.99%	2
South Africa	0.99%	2
Switzerland	0.99%	2
Tunisia	0.99%	2
Türkiye	0.99%	2
Viet Nam	0.99%	2
Albania	0.50%	1
Antigua and Barbuda	0.50%	1
Azerbaijan	0.50%	1
Belgium	0.50%	1
China	0.50%	1
Cyprus	0.50%	1
Guatemala	0.50%	1
Iraq	0.50%	1
Ireland	0.50%	1
Israel	0.50%	1
Korea (Republic of)	0.50%	1
Kosovo Republic of	0.50%	1
Kyrgyzstan	0.50%	1
Lebanon	0.50%	1
Lithuania	0.50%	1
Malta	0.50%	1
Norway	0.50%	1
Panama	0.50%	1
Peru	0.50%	1
Portugal	0.50%	1
Russian Federation	0.50%	1
Tanzania, United Republic of	0.50%	1
Thailand	0.50%	1
Trinidad and Tobago	0.50%	1

United Arab Emirates	0.50%	1
Uruguay	0.50%	1
Yemen	0.50%	1
TOTAL		202

Q45 Which race or ethnicity best describes you? Please choose only one.

Answered: 208 Skipped: 82

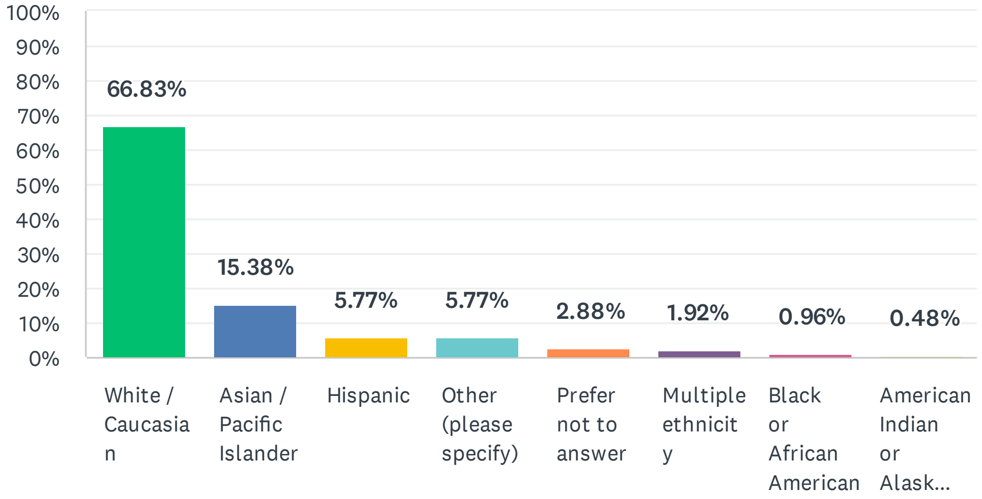

ANSWER CHOICES	RESPONSES	
White / Caucasian	66.83%	139
Asian / Pacific Islander	15.38%	32
Hispanic	5.77%	12
Other (please specify)	5.77%	12
Prefer not to answer	2.88%	6
Multiple ethnicity	1.92%	4
Black or African American	0.96%	2
American Indian or Alaskan Native	0.48%	1
TOTAL		208

#	OTHER (PLEASE SPECIFY)	DATE
1	south asian	6/13/2024 9:35 AM
2	Arabic	5/18/2024 12:04 AM
3	Native argentinian - inmigrant europe	5/8/2024 1:40 PM
4	Chinese	5/6/2024 9:30 PM
5	South East Asian (Vietnamese)	5/6/2024 1:38 PM
6	Malay	5/6/2024 1:12 PM
7	I am Armenian	5/6/2024 11:29 AM
8	north indian	5/6/2024 10:58 AM
9	Turkish	5/6/2024 10:20 AM
10	Arab	5/6/2024 10:14 AM

11	North african	5/6/2024 10:13 AM
12	Indonesia	5/6/2024 10:10 AM
